# Supplementary material for: Optimized whole-genome CRISPR interference screens identify ARID1A-dependent growth regulators in human induced pluripotent stem cells
Source: Stem Cell Reports. 2023 Apr 6;18(5):1061–74. doi: 10.1016/j.stemcr.2023.03.008 (PMC10202655; doi:10.1016/j.stemcr.2023.03.008)
Supplement: Document S1. Supplemental experimental procedures, Figures S1–S7, Table S4, and Note S1 [file mmc1.pdf]

Stem Cell Reports, Volume *18*

Supplemental Information

Optimized whole-genome CRISPR interference screens identify ARID1A-dependent growth regulators in human induced pluripotent stem cells

Sunay Usluer, Pille Hallast, Luca Crepaldi, Yan Zhou, Katie Urgo, Cansu Dincer, Jing Su, Guillaume Noell, Kaur Alasoo, Omar El Garwany, Sebastian S. Gerety, Ben Newman, Oliver M. Dovey, and Leopold Parts

INDEX

Supplemental FIGURES 2

Supplemental Figure 1. 2

Supplemental Figure 2. 3

Supplemental Figure 3. 4

Supplemental Figure 4. 5

Supplemental Figure 5. 7

Supplemental Figure 6. 8

Supplemental Figure 7. 9

Supplemental Note 10

Supplemental Experimental Procedures 11

Supplemental Table 4: Primer sequences 16

Supplemental FIGURES

Supplemental Figure 1.

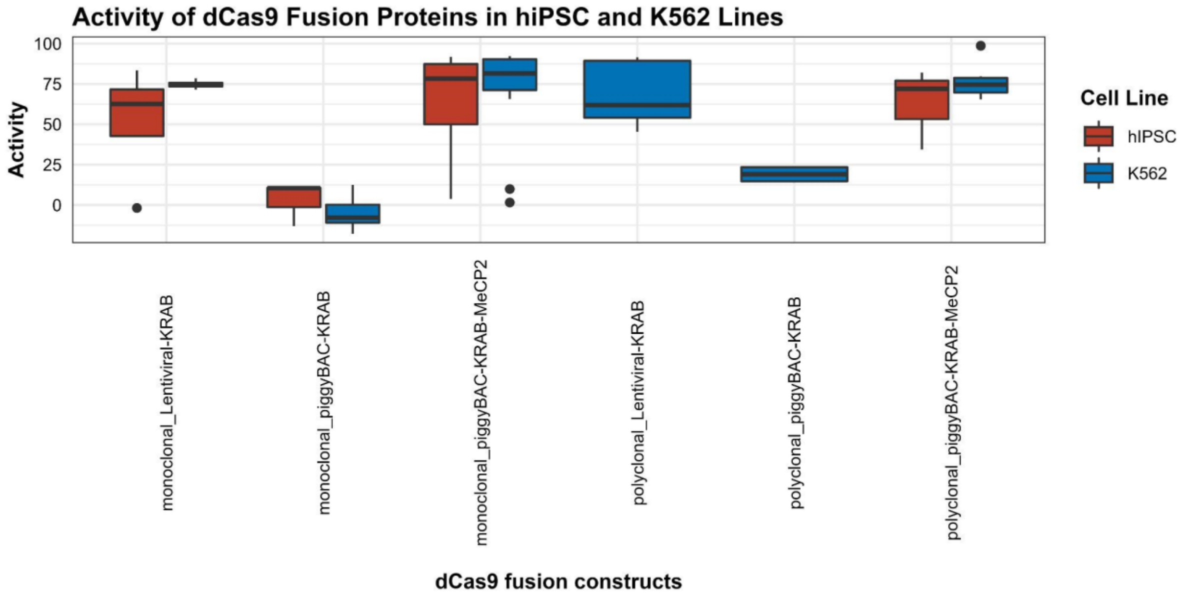

Supplemental Figure 1 – Related to Figure 1.C: Repression activity (y-axis) for different constructs and delivery methods (x-axis) into human iPSCs (red) and K562 cells (blue) for different mono- and polyclones (box and whiskers).

Supplemental Figure 2.

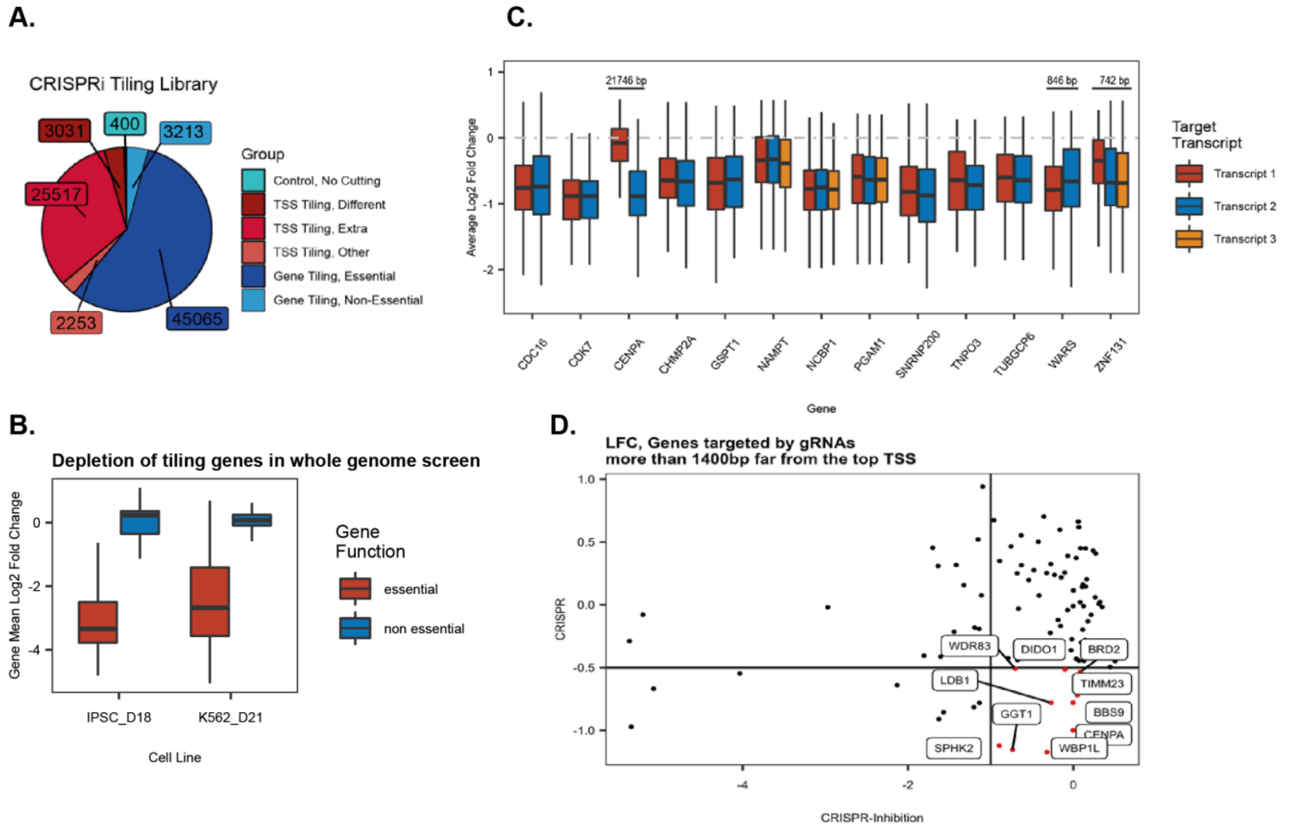

Supplemental Figure 2-(Related to Figure 2): **A.** Composition of CRISPRi tiling guide RNA library used in Figure 2. Numbers in boxes: guide RNA numbers in each sub-library. Teal: control library with no genomic targets. Red shades: transcription start site tiling library (“TSS Tiling, Extra”) tiling from 0 to +100bp of TSS site for the single transcript of 882 genes essential in human iPSCs; different TSS tiling library (“TSS Tiling, Different”) tiling -200 to +300 bp of TSS site for the top two transcripts of 20 essential genes for which the TSS annotation in human iPSCs did not match the canonical one; other transcript tiling library (“TSS Tiling, Other”) tiling top two transcripts of 20 essential genes. Blue shades: gene tiling library targeting all protospacer adjacent motifs in coding sequence of a single transcript of 451 Hart essential genes (blue; Hart et al., 2014) and 36 non-essential genes (teal). Contents of the libraries are provided in Supplemental Table 1. **B.** Gene-mean log2-fold change (y-axis) of genes in the transcription start site tiling library (Figure 2) in whole genome screen in iPSCs and K562 lines (x-axis). Box: median and quartiles; whiskers: 95th percentile; red: core essential genes, blue: non-essential genes. **C.** Average log2-fold change (y-axis) for 13 genes (x-axis) with multiple TSSs. Colors: gRNAs targeting the alternative TSSs. **D.** CRISPRi log2-fold change (x-axis) compared to CRISPR log2-fold change (y-axis) for genes (markers) that have a top TSS that is at least 1.4kb away from the targeted one.

Supplemental Figure 3.

A.

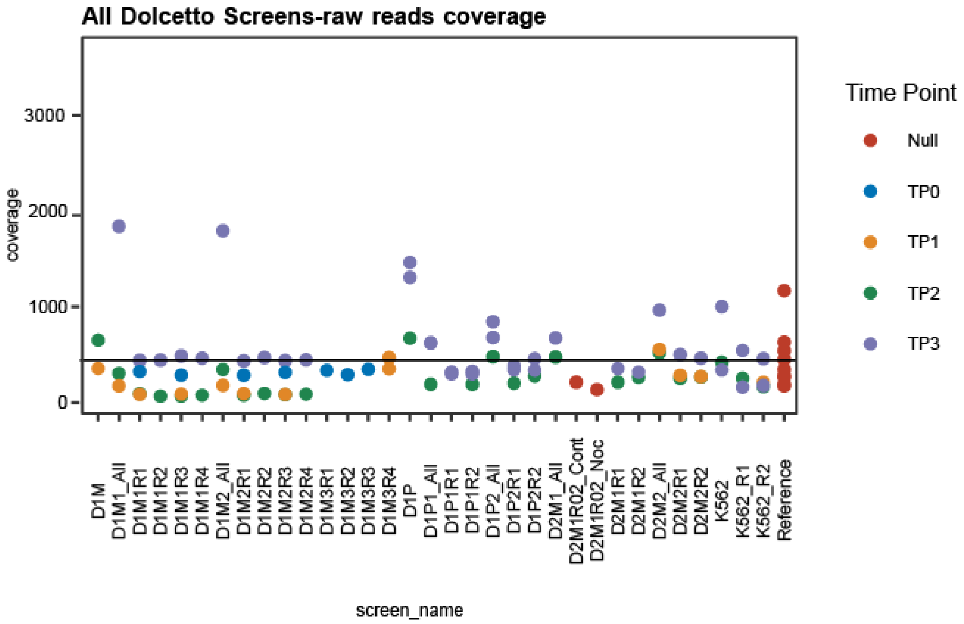

B.

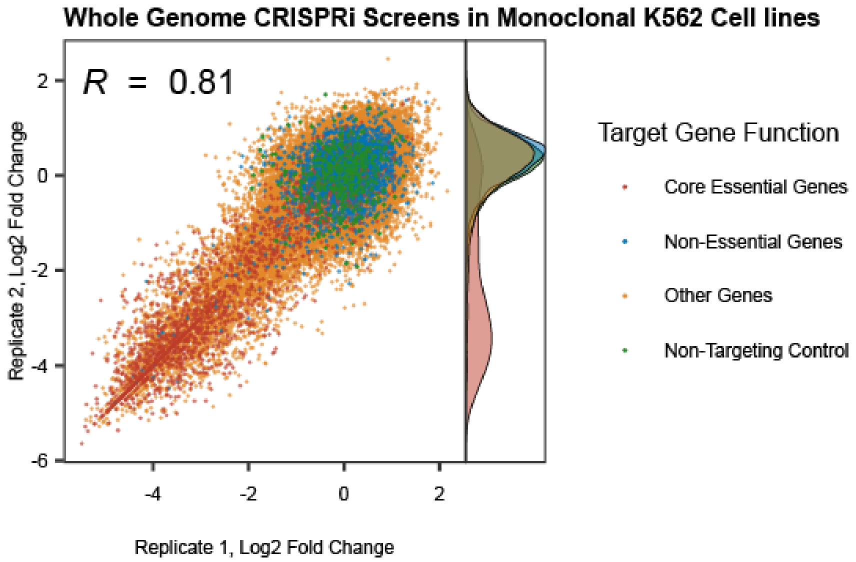

Supplemental Figure 3- (Related with Figure 3): **A.**Sequencing coverage (y-axis) of sequencing libraries (x-axis) of whole genome screens conducted using Dolcetto library (56,554 guide RNAs in total). Screen names; D: Donor, M: Monoclone, P: Polyclone, R: Replicate. Red: reference libraries, blue: timepoint 0 (days 3-5), yellow: timepoint 1 (days 9-11), green: timepoint 2 (days 13-15), purple: timepoint 3 (days 18-22), black line: average screen coverage (445X). **B.** Reproducibility of genome-wide screens in monoclonal K562 lines. gRNA log2-fold change in replicate 1 (y-axis) and replicate 2 (x-axis). Red: essential genes; blue: non-essential genes; yellow: other genes; green: non-targeting controls.

Supplemental Figure 4.

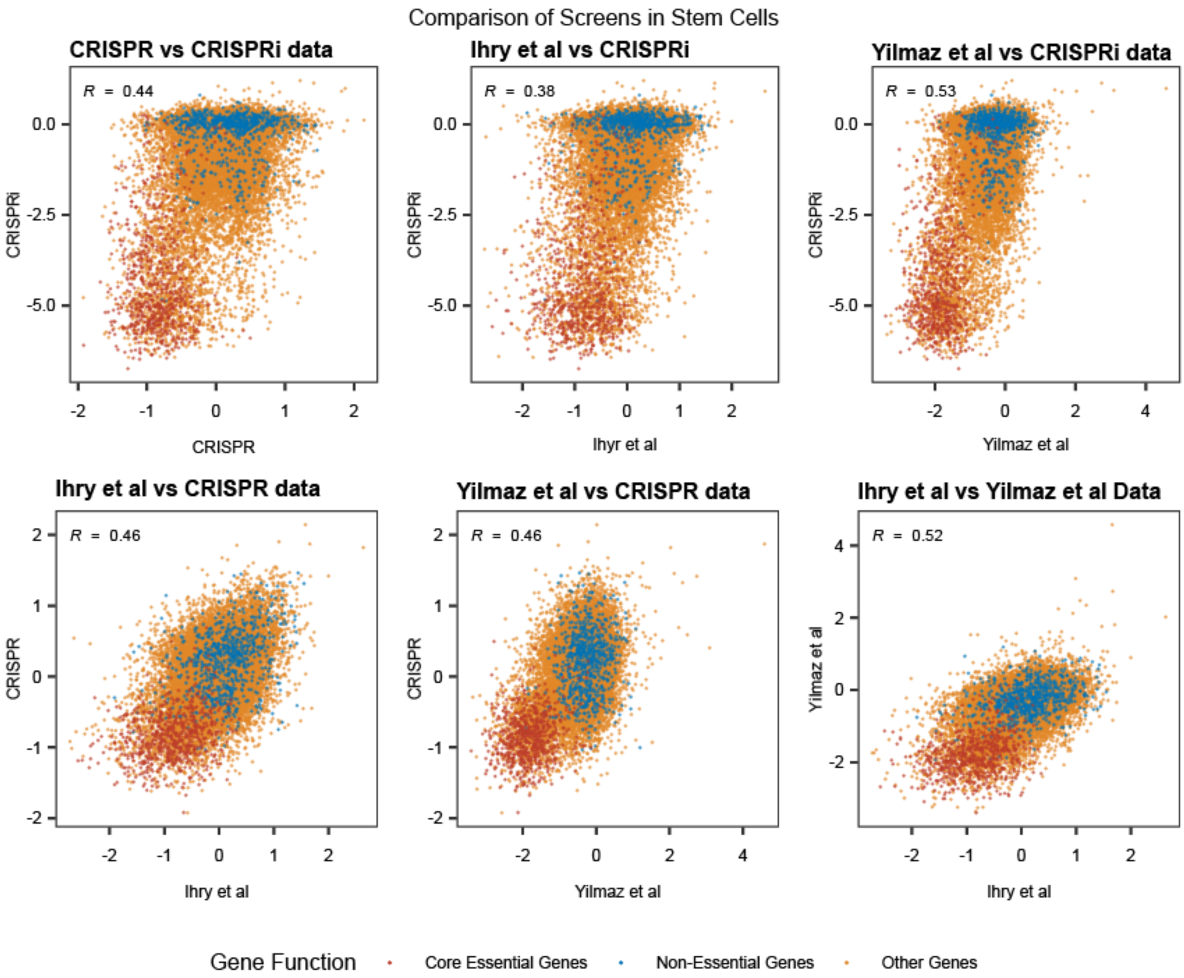

Supplemental Figure 4.A-(Related with Figure 4): Concordance of gene average log2-fold changes (x- and y-axes) of in-house CRISPR and CRISPRi screens in hiPSC with previously published CRISPR screens in diploid (Ihry *et al.*) and haploid (Yilmaz *et al.*) human embryonic stem cells (hESC). Red: essential genes; blue: non-essential genes; yellow: other genes. R: Pearson's correlation coefficient.

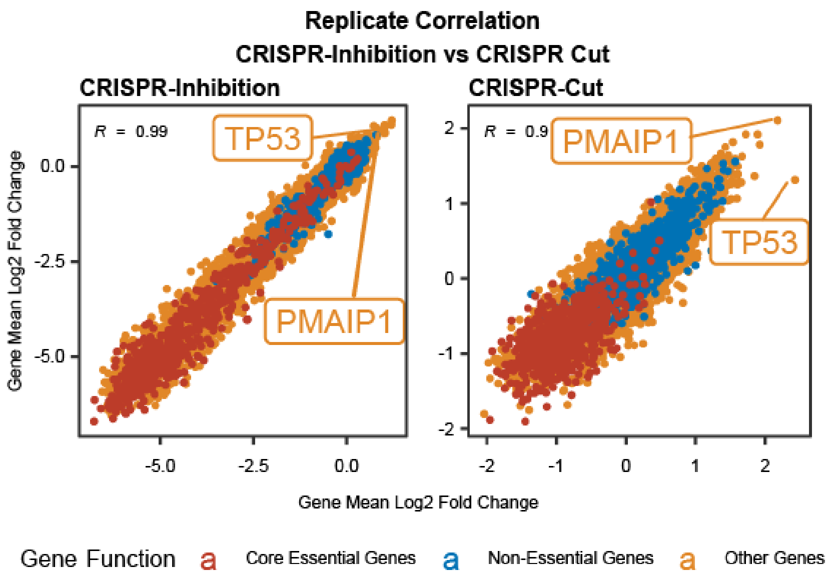

Supplemental Figure 4.B-(Related with Figure 4): Reproducibility and p53 effect in whole genome CRISPRi (left) and CRISPR (right) screens in monoclonal hiPSC lines. Gene mean log2 fold change value for replicate 1 (x-axis) and replicate 2 (y-axis). Data points for p53 and PMAIP1 genes are shown with an arrow. Red: essential genes; blue: non-essential genes; yellow: other genes.

Supplemental Figure 5.

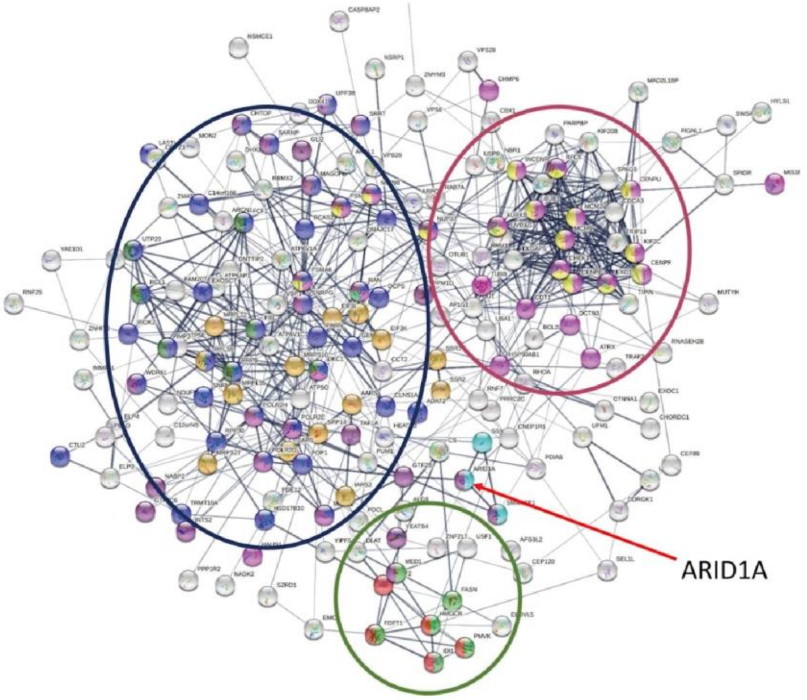

	Pathway	Description	Count in network	Strength	FDR
	HSA-8953854	Metabolism of RNA	41 of 659	0.82	4.3E-17
	HSA-1640170	Cell Cycle	30 of 647	0.69	1.3E-08
	HSA-72766	Translation	14 of 289	0.71	0.0012
	HSA-1655829	Regulation of cholesterol biosynthesis by SREBP (SREBF)	7 of 55	1.13	0.0015
	HSA-6790901	rRNA modification in the nucleus and cytosol	7 of 60	1.09	0.0022
	HSA-191273	Cholesterol biosynthesis	5 of 25	1.32	0.0049
	HSA-74160	Gene expression (Transcription)	31 of 1455	0.35	0.0124
	HSA-69620	Cell Cycle Checkpoints	16 of 269	0.8	5.7E-05
	GO:0071564	npBAF complex	3 of 12	1.42	0.0184

Supplemental Figure 5-(Related with Figure 5.E). STRING analysis of ARID1A. Large ellipses: manually curated clusters.

Supplemental Figure 6.

A.

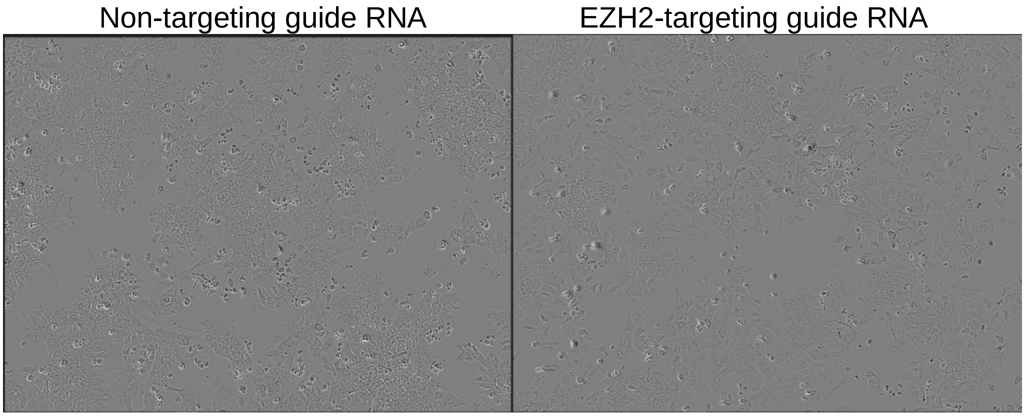

B.

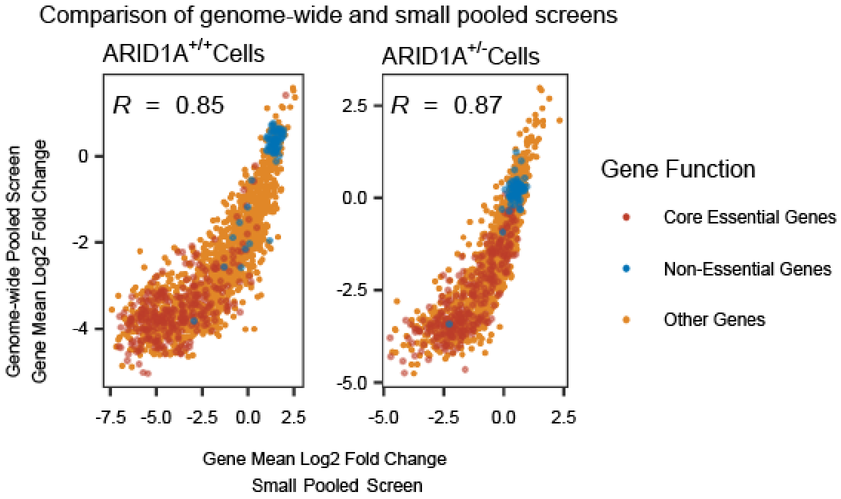

Supplemental Figure 6-(related with Figure 6). A. Microscope images from Incucyte live cell imaging system (10X objective) of ARID1A$^{+/+}$ hiPSCs infected with non-targeting guide RNA (left) and a guide RNA targeting EZH2 gene (right). **B.** Reproducibility of gene average log2-fold change (markers) in genome-wide (y-axis) and small-pooled screens (x-axis) in ARID1A$^{+/+}$ (left panel) and ARID1A$^{+/-}$ (right panel) hiPSC lines. Red: essential genes; blue: non-essential genes; yellow: other genes.

Supplemental Figure 7.

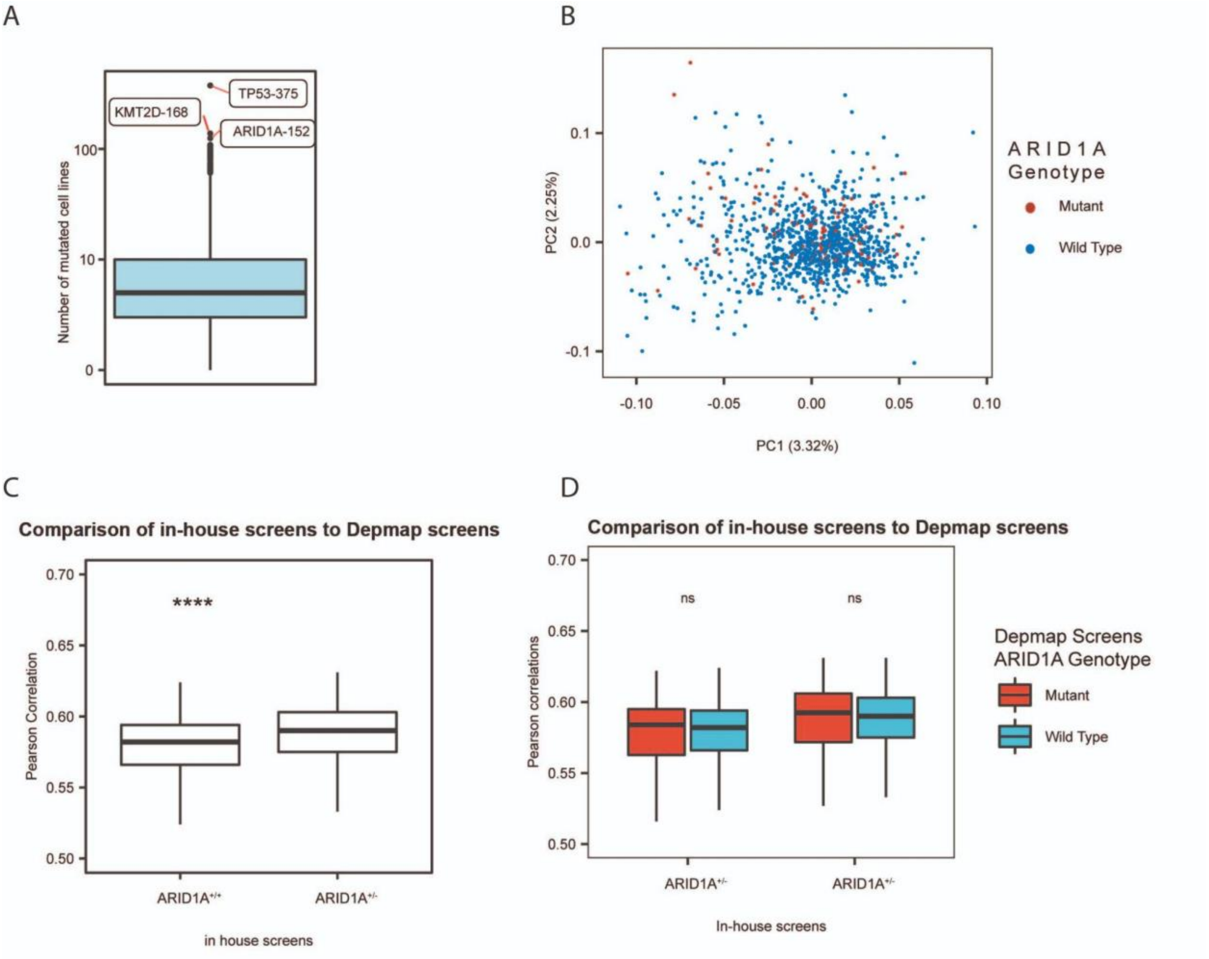

Supplemental Figure 7-(related with supplemental note): Comparison of in-house CRISPRi screens in ARID1A+/- and ARID1a+/+ hiPSC lines to DepMap screens. **A.** Number of mutated cell lines (y-axis) per gene. Labels: Gene and number of unique cancer lines with at least one mutation in a particular gene. Box: median and quartiles; whiskers: 95th percentile. **B.** PCA plot of DepMap CRISPR Gene Effect scores labeled according to ARID1A genotype of the cell line. Red: ARID1A mutant lines, Blue: ARID1A wild type lines. **C**. Correlation of in-house CRISPRi screens' Bayes Factor values to DepMap CRISPR Gene Effect scores. ARID1A$^{+/+}$: CRISPRi screen in ARID1A$^{+/+}$ hiPSC line, ARID1A$^{+/-}$: CRISPRi screen in ARID1A$^{+/-}$ hiPSC line. **D.** Correlation of in-house CRISPRi screens' Bayes Factor values to DepMap CRISPR Gene Effect scores separated by ARID1A genotype of DepMap cancer lines; Red: DepMap cancer lines with a mutation in ARID1A Gene. Blue: DepMap Cancer lines without ARID1A mutation. ARID1A$^{+/+}$: CRISPRi screen in ARID1A$^{+/+}$ hiPSC line, ARID1A$^{+/-}$: CRISPRi screen in ARID1A$^{+/-}$ hiPSC line.

Supplemental Note

Since our results in whole genome screens show gene interactions important for carcinogenesis, we asked if the sensitizing effect of the ARID1A mutation is similar to those of the damaging mutations in the cancer cell lines. To answer this question, we compared the survival of cancer cell lines upon gene perturbation in ARID1A wild-type and mutant contexts using data from the DepMap project (Dempster et al., 2021; Pacini et al., 2021). Among the 1,755 cancer lines in the database, 152 have at least one damaging mutation in the ARID1A gene (data: Q1/2022), making it one of the most mutated genes in this set of cancer lines (Supplemental Figure 7A). However, ARID1A mutation status was not associated with broad changes in gene essentiality in general (Supplemental Figure 7B). We did observe that gene essentiality estimates from our screen in the ARID1A+/- hiPSC line are significantly more correlated to the ones in cancer cell lines compared to the wild-type line (Supplemental Figure 7C). Still, this effect is small, and the correlation is not affected by ARID1A mutation status of the cancer line (Supplemental Figure 7D).

Supplemental Experimental Procedures

Lentivirus production. Lentivirus preps were produced in 293FT cells transfected with lentiviral delivery vectors together with second generation packaging system consisting of psPax2 (Addgene 12260), and pMD2.G (Addgene 12259) using Lipofectamine LTX with plus reagent (ThermoFisher 15338-030). 293FT cells were seeded on gelatin-coated 10cm culture dishes and grew until 80% confluent. On the day of transfection, 293FT medium was replaced by fresh medium. To prepare the transfection mix, 5.4 µg of a lentiviral vector, 5.4 µg of psPax2 (Addgene 12260), 1.2 µg of pMD2.G (Addgene 12259) and 12 µl of PLUS reagent were added to 3 ml of Opti-MEM Reduced Serum Media (ThermoFisher 31985-062) and incubated for 5 minutes. After addition of 36 µl of LTX reagent the mixture was incubated for another 30 minutes. The transfection mixture was added dropwise on top of 293FT cells and incubated at 37°C. After 48 hours, the medium with viral particles was collected and fresh medium was added on top of cells. After 24 hours, virus particles were harvested for the second time with medium, and added to the first harvest. All of the harvested virus was filtered using 0.45 um SFCA syringe filter (Nallgene, 190-2545), aliquoted and stored at -80ºC.

hIPSC infection. hiPSC cells were harvested using accutase (Stemcell technologies, 07920) and resuspended in mTeSR-E8 Media (Stemcell technologies, 05990) supplemented with 10µM Rock inhibitor (Ri) Y-27632 (Stemcell technologies, 72304). 0.5M cells were aliquoted and mixed with 200µl virus suspension and seeded on one well of 6 well-plate (in replicate). After 24 hours medium with Rock inhibitor was removed and fresh medium without Ri was added on top of cells. After two days, the medium was changed with mTeSR-E8 medium supplemented with 10 µg/ml Blasticidin (TOKU-E, B001), and selection was continued for 10 days.

K562 infection. $2x10^6$ K562 cells were aliquoted into 10 ml supplemented RPMI medium in duplicate. 100 µl of virus suspension and Polybrene (hexadimethrine bromide, Sigma) at a final concentration of 8 µg/ml were added, and the cell suspensions were centrifuged at 1000g for 30 minutes, resuspended, and seeded in 75 ml culture flasks with an additional 15 ml top up with supplemented RPMI media. After 48 hours, cells were passaged into media supplemented with 15 µg/ml Blasticidin (TOKU-E, B001), and selection was continued for 10 days.

PiggyBac transposition for hiPSCs. hiPSCs were transfected with

pB-CAGGS-dCas9-KRAB-MeCP2 or pPB-dCas9-KRAB vectors together with mPBase transposase vector using TransIT®-LT1 Transfection Reagent (Mirus Bio,MIR2300) as recommended by the producer. In summary, cells were harvested with accutase, and resuspended in mTeSR-E8 media supplemented with 10µM Rock inhibitor (Ri) Y-27632 (Stemcell technologies, 72304). Prepare 0.5M cell/ml cell suspension in mTeSR+E8 +Ri media. 400 µl of Opti-MEM Reduced Serum Media (ThermoFisher 31985-062) was aliquoted in a tube and 4µg plasmid DNA (1.8 µg delivery vector, 1.8 µg mPBase, 0.4µg pCS2-GFP) was added. The transfection mixture was incubated at room temperature for 20 minutes, and added onto one well of 6-well plate (Corning, 3516) with 0.5ml of mTeSR-E8+Ri media. After incubating the transfection mix on the plate for another 5 minutes, 1ml of $0.5x10^6$ cell/ml cell suspension was added on top. After 24 hours, the medium was changed to remove Rock inhibitor, and after 48 hours, the medium was changed to mTeSR-E8 supplemented with 10 µg/ml Blasticidin (TOKU-E, B001). Antibiotic selection continued for 10 days.

PiggyBac transposition for K562 cells. Cells were transfected with

pB-CAGGS-dCas9-KRAB-MeCP2 or pPB-dCas9-KRAB vectors together with mPBase transposase vector using Lipofectamine LTX reagent as described above for 293FT cells. pCS2-GFP vector was used as transfection control and the ratio of delivery vector:

mPBas:pCS2-GFP was 1:1:0.2. Blasticidin selection started after 2 days and continued for 10 days.

Cloning the reporter system

To measure CRISPRi efficacy, we devised a reporter system expressing BFP, GFP, and a guide RNA targeting GFP promoter from the same transient expression vector (Figure 1A). First, the pCS2-GFP vector was linearised with HindIII, and a gBlock (#639) with guide RNA target site was cloned into the GFP promoter region with Gibson assembly (Gibson et al., 2009) to obtain pCS2_CMV_gRNAtarget_GFP vector as a backbone. Meanwhile, the pU6-gRNA5 vector was linearised with BbsI, and oligos with target (#641) and mock (#640) gRNA were cloned after the U6 promoter with Gibson assembly. The U6+gRNA block was amplified from this vector (primers #642 and #643) and cloned into the SapI digested pCS2-BFP vector. The U6+gRNA+BFP block was then amplified from this vector (primers #646 and #647), the PGK promoter was amplified from pKLV2-U6gRNA5(gGFP5)-PGKBFPGFP-W vector (primers #644 and #645), and both cloned into the 5.2kb backbone of pCS2>CMV-GFP-SV40pA><CMV-mRuby2-bGHpA< vector linearised with EcoRi+MluI with Gibson assembly to produce pCS2-iREP-GFP-PGK-BFP-U6-gRNA-iRep or pCS2-iREP-GFP-PGK-BFP-U6-gRNA-mock vectors (all in one reporter system). All Gibson assembly reactions were conducted using NEB Gibson assembly master mix (E2611L) according to the manufacturer's protocol. Assembled constructs were purified using Monarch PCR purification kit (NEB, T1030S) and used to electroporate into electrocompetent bacteria (NEB, C3020K) according to the manufacturer’s instructions.

Generating the CRISPRi tiling library

Cloning and titration. sgRNAs were synthesized as complex oligonucleotide pools with several sub-pools (Genscript). Subpools were amplified with PCR, and Gibson assembly homology sequences were added to sgRNA sequences in a second round of amplification (primers #745 and #746). The lentiviral backbone vector pKLV2-U6gRNA5(BbsI)-ccdb-PGKpuroBFP-W (AddGene: 67974) (Tzelepis et al., 2016) was linearised with BbsI. Amplified sgRNA pools were inserted into the vector backbone with Gibson assembly reaction using NEB Gibson assembly master mix (E2611L) according to the manufacturer's protocol. Assembled constructs were purified using Monarch PCR purification kit (NEB, T1030S). The assembled plasmid library was electroporated into electrocompetent bacteria (NEB, C3020K) according to the manufacturer’s instructions as 3 reactions. 5µl of recovered bacteria was diluted 1:10 three times, and dilutions were seeded on ampicillin plates, while the remaining bacteria were seeded in liquid culture with ampicillin selection (100 µg/ml). The next day, bacterial colonies on agar plates were counted to calculate the coverage of the library, which was between 80x and 100x. The plasmid library pool was isolated from overnight bacteria culture using QIAGEN HiSpeed Plasmid Midi (small pooled library) of maxi (TSS tiling library) kits (QIAGEN, 12643 and 12662). Lentivirus was produced from cloned plasmid pools in 293FT cells as described above. To determine virus titer hiPSC-(Fiaj-1) dCas9-KRAB-MeCP2 cells were suspended as 0.16M cells/ml suspension. 5 x 2.5 ml of cell suspensions were mixed with five different amounts of virus between 20µl to 100µl. 1ml of each virus cell mixture was seeded one well of vitronectin pre-coated 12 well plate. After 3 days, cells were collected with accutase, washed, and resuspended in FACS buffer (PBS with 2% FBS) and analysed by FACS (Cytoflex, BD). Best fit line formula of percentage BFP positive cells against virus volume was used to calculate the volume of virus required for 0.3 multiplicity of infection (MOI).

Reamplification and titration of the Dolcetto CRISPRi library

Dolcetto library (Sanson et al., 2018) was acquired from Addgene (#1000000114) as two plasmid pools, each with a half-library (Set A and B) in the XPR_500 backbone. Only Dolcetto Set A plasmid pool was used in this study. 350 ng Dolcetto setA library pool was used to transform 100 µl electrocompetent bacteria (NEB, C3020K), split into 4 x 25 µl

electroporations. Electroporated bacteria were recovered in 3ml recovery media and all 4 cultures were mixed together. 5 ul of the culture mix was diluted 1:10 serial diluted 5 times and each dilution seeded on LB agar+ Amp plates. Remaining transformed bacteria culture seeded in 0.5ml LB+ Ampicillin (100 µg/ml) was left overnight. Plasmid library pool was isolated from overnight culture using HiSpeed maxi plasmid isolation kit (QIAGEN, 12662). Lentivirus was produced from the amplified plasmid pool as described above. To titrate the virus for hiPSCs, hIPSC+dCas9_KRAB+MeCP2 lines were collected with accutase and 1 M cells were resuspended in mTeSR-Plus +Ri media at 1.3x10^5 cells/ml. Different volumes of virus prep from 0 µl to 1.6µl were mixed with mTeSR-Plus +Ri media up to 600 µl. 600 µl of cell suspension was added on top of each virus dilution and mixed. 50 µl of each virus cell mixture were seeded into 10 wells of each of 96-well plates, resulting in two plates with 6 different virus/cell ratios. On day 2 post-infection, the medium in one plate was changed with mTeSR-Plus, and in the other plate with mTeSR-Plus + Puromycin (0.5 µg/ml). On day 4 post-infection, the medium in each well was replaced by 100µl mTeSR-Plus medium + 20µl MTS dye (Promega, G3582) to compare living cell amounts between plates. The plates were incubated at 37 C for 2 hours, followed by the addition of 25µl of 10% SDS solution on each well. Plates were analyzed in Multiskan™ GO Microplate Spectrophotometer plate reader (Thermo Scientific, 51119200) at wavelengths of 490 nm and 700 nm. Raw reads were adjusted after blank and background (700 nm) readings were subtracted from target readings (490 nm). Virus titer was determined from the ratio of puromycin treated and untreated cells. To titrate the virus for K562 cells, the cells were collected, counted, and aliquoted to have 50,000 cells per well, 12 wells for each of 6 virus titers. Different amounts of virus prep were mixed with cell suspensions and distributed on a 96-well plate. Each well was topped up with supplemented RPMI media + Polybrene (8ug/ml final concentration) up to 150 ul. Plates were centrifuged at 1000 rcf for 30 minutes and resuspended. On day 3 after infection, cells were split into two 96-well plates. Cells in one plate were resuspended in supplemented RPMI media and the other plate in supplemented RPMI + puromycin (2µg/ml) media. On day 6 after the infection relative amounts of cells in each well were determined by MTS assay as described above.

Genomic DNA isolation and sequencing library preparation

Aliqouted cell pellets were thawed at room temperature and resuspended in 100 mM Tris-HCl, pH 8.0, 5 mM EDTA, 200 mM NaCl, 0.2% SDS and 1 mg/ml Proteinase K. After overnight incubation at 55oC, RNase was added on top of the cell suspension to the final concentration of 10 µg/µl, followed by 3h incubation at 37oC. Genomic DNA was precipitated with 100% isopropanol, spooled out, washed in 70% EtOH and air dried at room temperature. Following resuspension in TE buffer overnight, DNA was quantified with Quant-iT Broad Range kit (Q33130, ThermoFisher). gRNA cassettes were amplified from genomic DNA with two consecutive PCR reactions taking the target library coverage into account (used genomic DNA corresponding cell number for 500x for final timepoints, minimum 250x for interval timepoints). The first reaction with Q5 Hot Start High-Fidelity 2X Master Mix (NEB) amplifies the gRNA cassette (primers #1 and #2 for CRISPRi and small pooled screen libraries, primers #1 and #638 for Dolcetto library). PCR reactions were purified with QIAquick PCR Purification Kit (Qiagen, 28106) and quantified with nanodrop. Each library was diluted to 1ng/µl and used as template for the second PCR reaction where sequencing adaptors with index sequences were added as described before (primers #15 and #NN indexing) (Tzelepis et al. 2016). PCR reactions were purified with 0.7X AMPure XP beads (Agencourt AMPure XP beads; Beckman, Cat.no. A63881), quantified with Quant-it High Sensitivity Kit (Q33120, ThermoFisher), pooled and single end sequenced using primer #16.

Arrayed gRNA cloning, lentivirus production

Cloning. Each guide RNA was ordered as top and bottom strand oligos mixed in single well creating double stranded DNA with cloning overhangs (Sigma Aldrich, Supplemental Table 4).

Oligos were phosphorylated with T4 Polynucleotide Kinase (NEB M0201) at 37oC for 30 minutes. pKLV2-U6gRNA5(BbsI)-PGKpuro2AmCherry-W (Addgene 67977) (Tzelepis et al. 2016) was linearised with BbsI at 37oC overnight. Phosphorylated gRNA oligos were cloned into linearized backbone with T4 DNA ligase (NEB M0202*) by incubating 1h at room temperature. 3 µl of the ligation products were used to transform 20µl of zymo mix&go 10B competent cells (Zymo Research, T3020). Transformed cells were grown in 2ml 2XLB media overnight. gRNA plasmids were isolated using QIAprep Turbo Miniprep Kit (Qiagen, 27191). gRNA sequences were confirmed by Sanger sequencing.

Lentivirus production. Flat bottom 96 well plates were coated with 0.1% gelatin, and 293T cells were seeded as 20K cells/well. 0.1ug LV transfer plasmid, 0.1ug psPAX2, and 0.02 ug pMD2.G were used to transfect cells with Lipofectamine LTX Reagent (15338100, Thermo Fisher) by scaling manufacturer's protocol. Cultures were collected from each well after 48 hours, centrifuged at 500 rcf for 20 minutes, and supernatant was collected carefully without disturbing the pellet.

Cell phenotyping

Comparison of WT and ARID1A$^{+/-}$ lines. Wild type and ARID1A$^{+/-}$ Kolf hiPSC lines were seeded at 20,000/cm2 density in 12-well plates (6 well each) in mTeSR Plus medium supplemented with Rock inhibitor (Ri). The next day, medium was changed to remove Ri and the plate was placed in Incucyte S3 live imaging system (Sartorius) and imaged every 2 hours for 50 hours using 10x objective taking 16 frames per well. The growth rate was measured as percent confluency and normalized to the first time point.

Oprozomib treatment measurements. Wild type and ARID1A$^{+/-}$ Kolf hiPSC lines were seeded at 20,000/cm2 and 30,000/cm2 density respectively in 12 wells of 24 well plate. After 36 hours, both lines were at approximately 25% confluence. At this point, medium was replaced with mTeSR-Plus supplemented with different concentrations of Oprozomib (APExBIO, A1937) (10uM, 2uM, 0.4uM, 0.08uM, 0,02uM). The plate was then placed in the Incucyte live imaging system, and imaged every 2 hours for 26 hours using 10x objective taking 16 frames per well. At each time point, the growth rate was measured as percent confluency, and normalised to the first time point. Confluence was compared to no treatment wells at 32h after seeding.

CRISPR vs CRISPRi comparison. Monoclonal Fiaj-1_dCas9-KRAB-MeCP and Fiaj-1_Cas9 lines were collected, counted and infected with targeting and non-targeting guides (Supplemental Table 3). Infected cells were seeded on two wells of 24 plates and monitored in Incucyte S3 Live Cell imaging System with 10x objective taking 16 frames per well every two hours. Cell death was determined as the decrease in confluency after media change.

Growth assays with live imaging system. Cell growth was measured in Incucyte S3 Live Imaging System with confluence as a measure of cell number in different assays. Additionally, to validate confluence vs cell number correlation, cells were seeded in a 96 well plate at different densities and cell growth was measured by taking images every two hours with a 10X objective and 5 frames per well for 24h. After final measurement of confluence in the Incucyte live imaging system for each, we compared the optical density (OD) value obtained to the MTS assay and found it to be highly correlated.

.

Cell staining and FACS. Wild type and ARID1A+/- lines were infected with lentivirus with EZH2 targeting guides and seeded in 12 well plates. Infected cells were selected with puromycin for 9 days. Cells were collected with accutase and counted. 5x106 cells were aliquoted and washed once with FACS buffer (5% FBS in PBS). Cells were fixed in 1% PFA for 30 minutes at room temperature and blocked in FACS buffer for another 30 minutes. 1x106 cells were aliquoted in tubes and incubated with stem cell marker, perCP-CY-5.5-Mouse antihuman-TRA-1-60 antibody (BD Pharmingen, 561573) for 1 hour at room temperature.

HEK293T cells were used as a negative control for antibody sensitivity. Stained cells were analysed in CytoFLEX flow cytometer and with FlowJo analysis software (Beckman Coulter).

Supplemental Table 4: Primer sequences

Primer	Sequence	Name
#639	TGCCTGGGGACGTCGGAGCATACCTCATCAGGAACATGTTGGTGATTTAGGTGACACTATAGAATACAAGCT	Gibson-target-iREP
#640	GCTTTATATATCTTGTGGAAAGGACGAAACACCGGTTAAGCGACTTCGGCCAGGTTTAAGAGCTATGCTGGAAACAGCA	Gibson-mock-grna
#641	GCTTTATATATCTTGTGGAAAGGACGAAACACCGTACCTCATCAGGAACATGTGTTTAAGAGCTATGCTGGAAACAGCA	Gibson-iREP-gRNA
#642	GGGCCTCTTCGCTATTACGCCAGACGCGTCCAAGGTCGGGCA	GA-pCS2-U6
#643	ATACGCCATATTGAATTGGCTATGGTCGACACTAAAGGGAACAAAAGCGGATCC	GA-pCS2-scaffold
#644	CCGCCAGTGTGATGGATATCTGCAGTTAATTTAGCTTGTGCCCCAGTTTGCT	GA-BFP-bGHpA
#645	CTTCCTGCCCGACCTTGGACGCGTAATTCTACCGGGTAGGGGAGGCGC	GA-U6-PGK
745	GGCTTTATATATCTTGTGGAAAGGACGAAA	ssoligo-PCR-F
746	ACTTGCTATGCTGTTTCCAGCATAGCTCTT	ssoligo-PCR-R
#1	ACACTCTTTCCCTACACGACGCTCTTCCGATCTCTTGTGGAAAGGACGAAACA	Sequencing_gRNA_library_amplification
#2	TCGGCATTCCTGCTGAACCGCTCTTCCGATCTCTAAAGCGCATGCTCCAGAC	Sequencing_gRNA_library_amplification
#638	TCGGCATTCCTGCTGAACCGCTCTTCCGATCTTCTACTATTCTTTCCCCTGCACTGT	Dolcetto-lib-R
#15	AATGATACGGCGACCACCGAGATCTACACTCTTTCCCTACACGACGCTCTTCCGATCT	Sequencing_indexing_PCR_indexing
#NN	CAAGCAGAAGACGGCATACGAGATN11GAGATCGGTCTCGGCATTCCTGCTGAACCGCTCTTCCGATCT	Sequencing_indexing_PCR
#16	TCTTCCGATCTCTTGTGGAAAGGACGAAACACCG	Sequencing
