# Supplementary material for: Optimized whole-genome CRISPR interference screens identify ARID1A-dependent growth regulators in human induced pluripotent stem cells
Source: Stem Cell Reports. 2023 Apr 6;18(5):1061–74. doi: 10.1016/j.stemcr.2023.03.008 (PMC10202655; doi:10.1016/j.stemcr.2023.03.008)
Supplement: Document S2. Article plus supplemental information [file mmc5.pdf]

Stem Cell Reports
Article
OPEN ACCESS

Optimized whole-genome CRISPR interference screens identify ARID1A-dependent growth regulators in human induced pluripotent stem cells

Sunay Usluer,[1,*] Pille Hallast,[1] Luca Crepaldi,[1] Yan Zhou,[1] Katie Urgo,[1] Cansu Dincer,[1] Jing Su,[1] Guillaume Noell,[1] Kaur Alasoo,[2] Omar El Garwany,[1] Sebastian S. Gerety,[1] Ben Newman,[1] Oliver M. Dovey,[1] and Leopold Parts[1,2,*]
[1]Wellcome Sanger Institute, Cambridge, UK
[2]Department of Computer Science, University of Tartu, Tartu, Estonia
*Correspondence: sunay.usluer@sanger.ac.uk (S.U.), leopold.parts@sanger.ac.uk (L.P.)
https://doi.org/10.1016/j.stemcr.2023.03.008

SUMMARY

Perturbing expression is a powerful way to understand the role of individual genes, but can be challenging in important models. CRISPR-Cas screens in human induced pluripotent stem cells (iPSCs) are of limited efficiency due to DNA break-induced stress, while the less stressful silencing with an inactive Cas9 has been considered less effective so far. Here, we developed the dCas9-KRAB-MeCP2 fusion protein for screening in iPSCs from multiple donors. We found silencing in a 200 bp window around the transcription start site in polyclonal pools to be as effective as using wild-type Cas9 for identifying essential genes, but with much reduced cell numbers. Whole-genome screens to identify ARID1A-dependent dosage sensitivity revealed the *PSMB2* gene, and enrichment of proteasome genes among the hits. This selective dependency was replicated with a proteasome inhibitor, indicating a targetable drug-gene interaction. Many more plausible targets in challenging cell models can be efficiently identified with our approach.

INTRODUCTION

Cell growth has to strike a balance between supporting development and survival across the lifespan against avoiding dysregulated proliferation. The genes required for this control have been mapped using genome-scale perturbation screens in different conditions and genetic backgrounds (Miles et al., 2016), and understanding them is important for restricting tumors as well as mitigating developmental disorders (Evers et al., 2016). There is an emerging consensus of the nature of gene essentiality from screens in many human cell lines, with a set of established core essential genes that are required for survival in most tested contexts, as well as a set of context-dependent vulnerabilities (Behan et al., 2019; Hart et al., 2015, 2017).

The CRISPR-Cas9 system has rapidly become the gold standard for pooled survival screens that collect this important information (Shalem et al., 2014; Wang et al., 2014). In brief, to knock out a gene, the Cas9 protein is directed to it by a guide RNA (gRNA), which results in a double-stranded DNA break that is ultimately repaired by error-prone pathways leading to small insertions and deletions that often disrupt the reading frame (Mali et al., 2013). These perturbations are relatively straightforward to parallelize, which enables efficient screening (Hanna and Doench, 2020). However, the DNA break generated by Cas9 can be toxic and trigger cell death, especially in stem cell contexts (Aguirre et al., 2016; Haapaniemi et al., 2018; Ihry et al., 2018; Peets et al., 2019; Rosenbluh et al., 2017). An alternative approach that does not suffer from this limitation is CRISPR inhibition (CRISPRi) (Gilbert et al., 2013; Qi et al., 2013), which employs a catalytically inactive Cas9 (dCas9) protein that is unable to cut DNA, and is usually fused to different effector domains to improve inhibition (Alerasool et al., 2020; Yeo et al., 2018). CRISPRi efficacy is known to be dependent on targeting the right transcript at the right distance to the transcription start site (TSS), but also to vary across contexts, which motivates evaluating and establishing these dependencies in the extensively used systems (Radzisheuskaya et al., 2016; Rosenbluh et al., 2017; Sanson et al., 2018; Yeo et al., 2018).

An important context to study disease mechanisms, drug targets, and development is stem cells, and in particular induced pluripotent stem cells (iPSCs) (Liu et al., 2020; Przybyla and Gilbert, 2022). The first genome-wide CRISPR-Cas screens in these cells have already shed light on mechanisms regulating embryonic stem cell survival and growth (Ihry et al., 2019; Mair et al., 2019; Peets et al., 2019). Cell types derived from stem cells via differentiation have been used to effectively and reversibly silence endogenous genes in cardiomyocytes (Mandegar et al., 2016), as well as to measure gene essentiality in neurons (Tian et al., 2019). Edited human iPSCs (hiPSCs) were also useful to identify early events of carcinogenesis before the accumulation of secondary mutations frequently observed in cancer lines (Wang et al., 2021). However, the potential of large-scale screening in stem cells is severely hampered by their cost, as the medium is expensive, and the cell death due to standard Cas9 action leads to requirements of very large numbers of cells. To unleash this potential, cost-permissive, large-scale, effective dropout CRISPRi screens have to be established in iPSCs, which requires

This is an open access article under the CC BY license (http://creativecommons.org/licenses/by/4.0/).

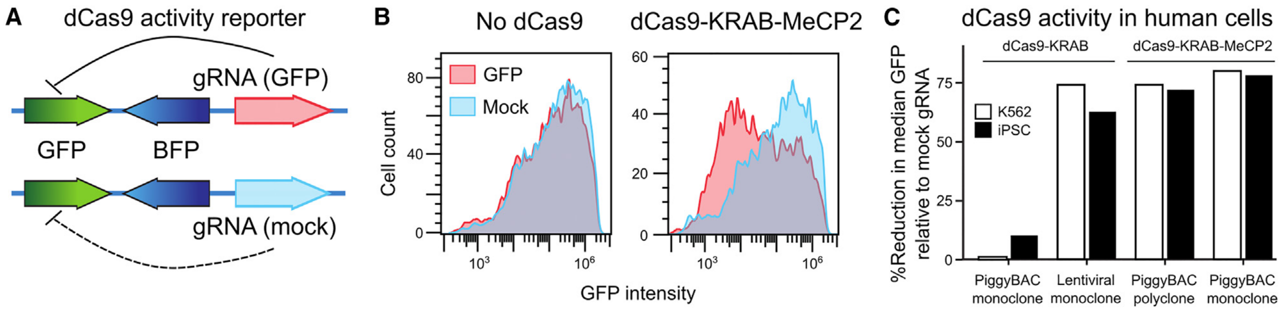

Figure 1. Identifying potent CRISPRi reagents in hiPSCs

(A) Design of a dCas9 activity reporter based on internal BFP control, and GFP-targeting gRNA comparison against a mock gRNA.

(B) Frequency (y axis) of GFP intensity (x axis) for GFP-targeting gRNA (red) and mock control (blue) for dCas9-negative cells (left panel) and dCas9-KRAB-MeCP2-positive cells (right panel).

(C) dCas9 construct activity, quantified as the fraction of median GFP abundance with GFP-targeting gRNA compared with mock gRNA in BFP-positive cells (y axis) for two different dCas9 constructs of different clonality and delivery method (x axis) in K562 cells (white bars) and iPSCs (black bars). Results are the median of at least three independent monoclones or tests.

understanding the efficacy of effector proteins and targeting constructs in this system. The current proof-of-principle studies are not yet informative enough for choosing the parameters for a screening campaign.

Here, we develop an effective CRISPRi screening approach in iPSCs, and use it to map mutation-specific and drug-sensitive genetic dependencies. First, we compare multiple fusion proteins, and identify dCas9-KRAB-MeCP2 as the most potent one. We then establish the characteristics of successful gRNAs that effectively repress their target using this fusion, identifying both the activity window as well as highlighting the importance of correct transcript selection. Next, we conduct whole-genome screens to determine the coverage requirements for an effective dropout screen in iPSCs, and compare these to standard CRISPR screens. Finally, we demonstrate the efficiency of CRISPRi screens to identify nocodazole-dependent gene essentiality, as well as genes that impact growth differently as a result of a mutation to the *ARID1A* cancer and developmental disorder gene.

RESULTS

Effective gene silencing with CRISPRi depends on the fusion protein used and the choice of gRNAs. Therefore, we first explored the potency of dCas9 alternatives in hiPSCs, and identified the effect of gRNA features, such as proximity to different TSSs.

A rapid gene silencing efficacy test identifies the dCas9-KRAB-MeCP2 fusion protein as the most potent in hiPSCs

To identify the most potent dCas9 fusion construct, we compared three previously reported versions: dCas9-KRAB with piggyBAC delivery, KRAB-dCas9 with lentiviral delivery, and dCas9-KRAB-MeCP2 with piggyBAC delivery (Yeo et al., 2018). We produced polyclonal and monoclonal lines in iPSCs, and the human myeloid leukemia cell line K562 for each, and measured their efficacy in a dual fluorescence reporter system (Figures 1A, 1B, and S1). As expected, we observed reduced intensity of the targeted GFP gene in nearly all cases, with the most effective monoclonal lines outperforming the polyclonal ones (Figure 1C). In both K562 and iPSCs, the most successful clone was a monoclonal line with the dCas9-KRAB-MeCP2 construct, so we chose these lines for our screening applications, and retained the polyclones for reference as well.

dCas9-KRAB-MeCP2 silencing window can extend to 1.4 kb

After picking the fusion protein to work with, we characterized the determinants of successful gene repression with dCas9-KRAB-MeCP2 in iPSCs, starting with TSS distance. To determine the optimal target window, we designed a diverse gRNA library against various non-coding regions (including alternative TSSs) and coding sequences (Figure S2A).

We first targeted all NGG protospacer-adjacent motifs (PAMs) in 100 base pairs (bp) downstream of the TSS for 252 essential genes. On average, downregulation was most potent in the 20–40 bp downstream window (mean $\log_2$ fold change = –2.5), with a small but significant decrease of efficacy elsewhere (weakest mean $\log_2$ fold change = –2.2; Figure 2A). We next tiled all PAMs in a broader window of –200 to 300 bp around the TSS for 17 essential genes. We observed the strongest downregulation in the 0 to 100 bp window just downstream of the TSS, but more tempered effects

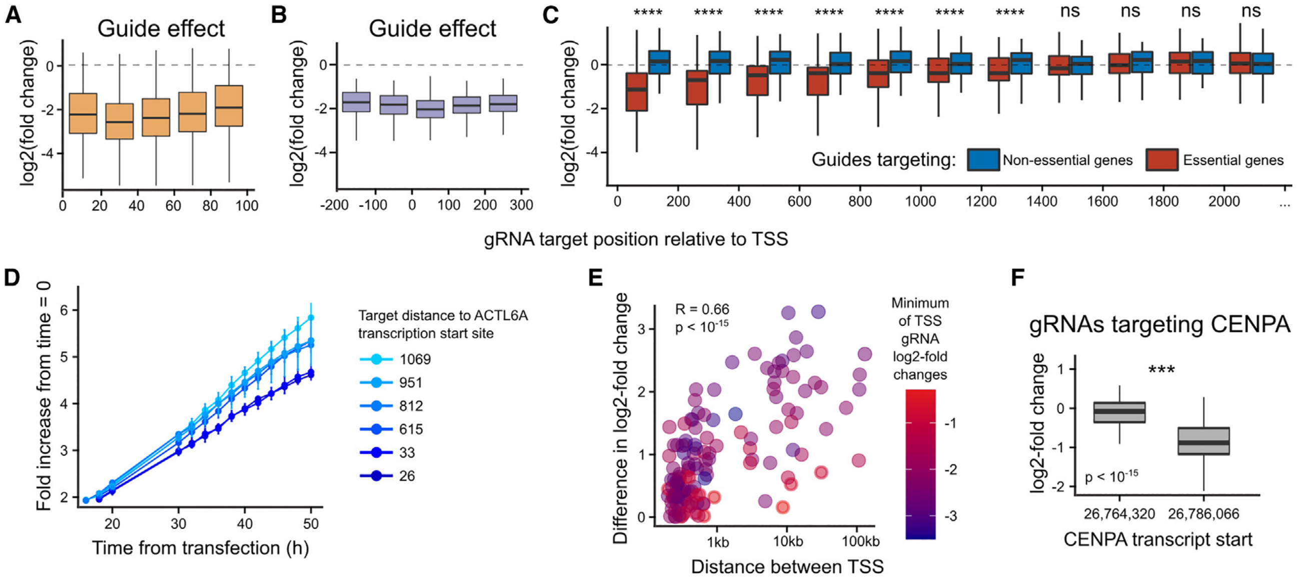

Figure 2. TSS targeting range and annotation effect on CRISPRi efficiency

A) gRNA log_2 fold change in frequency (y axis) at different positions relative to the transcription start site (TSS) (x axis). Box, median and quartiles; whiskers, 95th percentile; dashed line, no effect. Data for 6622 gRNAs targeting 252 essential genes.
(B) As (A), but data for 1,816 gRNAs targeting 17 essential genes in a different range.
(C) As (A), but data for 45,065 gRNAs targeting 451 essential genes (red) and 4,122 gRNAs targeting 49 non-essential genes (blue) in a different range. Wilcoxon test $^{****}p < 10^{-5}$; ns, not significant.
(D) Fold increase in confluency from time of transfection (y axis) across time (x axis) for gRNAs targeting *ACTL6A* at different distances to its TSS (colors).
(E) Guides targeting distant TSSs of the same gene have different effects. Difference in average log_2 fold change of guides targeting two transcripts (y axis) according to TSS separation (x axis). Color: stronger of the two transcripts' downregulation phenotypes.
(F) Log_2 fold change of gRNAs targeting *CENPA* gene (y axis) for gRNAs targeting two different TSSs (x axis). Box and whiskers as (A). Wilcoxon test $^{***}p < 10^{-4}$

further away (mean log_2 fold change −2.05 vs. −1.81; Figure 2B).

Given the relative evenness of guide efficacy in these windows close to the TSS, we finally tested even more distal targets. We re-used a design that tiled coding sequences of 440 essential genes depleted in a genome-wide screen (Figure S2B) and observed significant depletion of at least 25% as far as 1.4 kb from the TSS, with effects further out indistinguishable from controls (Figure 2C). The decrease of efficacy was gradual, with an average log_2 fold change for this library reducing from −1.2 within 100 bp of the TSS to −0.4 at 1.2–1.4 kb away. To validate the distance dependence of depletion phenotypes, we cloned six gRNAs targeting the essential *ACTL6A* gene at distances to its TSS ranging from 26 to 1,069 bp, infected a monoclonal dCas9-KRAB-MeCP2 iPSC line with them, and monitored growth under a live imaging system. We observed a decrease in growth rate that was proportional to TSS proximity (Figure 2D), confirming that the phenotypic impact can be modulated in the cell population by varying the distance of the targeting guide to the TSS.

Targeting the correct TSS is important for CRISPRi efficiency

As the TSS can be cell-type dependent, picking the correct one is required for designing efficient CRISPRi reagents. To measure the impact of TSS annotation, we picked genes with two TSSs separated by at least 200 bp from the tiling experiment, and calculated the difference in mean log_2 fold change of the gRNAs targeting the two transcripts. We found that the CRISPRi phenotype change depended on the proximity of the two TSSs, with longer distances leading to larger differences in phenotype (Pearson's $R = 0.66$, $p < 10^{-15}$; Figure 2E).

Next we focused on 17 genes with alternative TSS annotation in iPSCs, with 13 of them showing minimum log_2 fold change below −0.5 at either of the TSSs, and three of the genes having alternative TSSs at least 500 bp apart. Only one gene (*CENPA*, median log_2 fold change 0.0 vs. −0.9), with more than 21 kb distance between transcripts, showed a differential repression effect, with the remaining 12 alternative TSSs efficiently targeted (Figures 2F and S2C). We then checked the TSS annotation

of the 4,203 genes showing a depletion phenotype in any previous essentiality screens (Funk et al., 2022), and expressed in hiPSCs according to the FANTOM database (Lizio et al., 2015). Only 94 of the top TSSs according to FANTOM are targeted by gRNAs that are more distant than 1,400 bp, and, of these, only 10 are depleted in CRISPR (<−0.5 LFC) but not CRISPRi (>−1 LFC) (Figure S2D), which also include the two we tested separately above. We provide alternative gRNA designs for all of them in Table S1. Overall, TSS annotation is important for CRISPRi efficiency with the KRAB-MeCP2 construct, and consistent with observations from tiling experiments above, especially when the transcripts are separated by more than 1 kb.

CRISPRi screening in iPSCs efficiently identifies essential genes

To measure the performance of the dCas9-KRAB-MeCP2 construct in identifying essential genes on the genome scale, we conducted screens in hiPSC and K562 cell lines with the Dolcetto library (Sanson et al., 2018). We aimed for 200× coverage during infection, maintained 500× coverage throughout the remainder of the screen across technical replicates, and had mean coverage of 443 for sequencing libraries (Figures 3A and S3A; experimental procedures).

The whole-genome CRISPRi dropout screens successfully identified essential genes in both iPSCs (Figure 3) and K562 cells (Figure S3B). The screens were highly reproducible between replicates and different iPSC lines (Pearson's R between gene average $\log_2$ fold changes >0.9; Figures 3B and 3C), and could successfully separate gold standard core essential and non-essential genes (Hart et al., 2014), with the area under the receiver-operator curve (AUC) above 0.9 in all cases (Figures 3D and 3E; Table S2). The performance at 50× coverage of a single replicate at day 18 post-infection was very close to that of a 200× screen combining four replicates (AUC = 0.977 vs. 0.978). There was little difference to another biological replicate (AUC = 0.978 vs. 0.980), as well as to combining information from multiple biological replicates (AUC = 0.980), or performing a single screen at 100× (AUC = 0.974). The separation of positive and negative controls increased with every passage, with the most performance gained when going from passage two at day 8 to passage three at day 14 (AUC = 0.957 vs. 0.977), and with little further improvement afterward (AUC = 0.980 at day 18). Altogether, 50× coverage screens in monoclonal lines measured at day 14 have near-optimal performance in iPSCs, but polyclonal lines and earlier timepoints can be used to trade quality for cost.

Producing monoclonal cell lines from a polyclonal pool requires at least 1 month of extra work and carries risks of additional positively selected mutations that can confound screening results. To test whether polyclonal cell populations suffice for high-quality screens, we conducted experiments in the parental polyclonal iPSC line with 70% silencing activity. We observed lower reproducibility of the biological replicates of polyclones (Pearson's R of gene mean gRNA LFC = 0.91 vs. 0.99 in monoclones), as well as worse resolution at 50× coverage to separate essential and non-essential genes (median AUC = 0.97 vs. 0.98 in monoclones). However, this performance is still above the range reported in large-scale resources such as the Cancer Dependency Map (Behan et al., 2019), with all of the lines screened with wild-type Cas9 showing AUCs below 0.95. In addition, the $\log_2$ fold changes are highly correlated between mono- and polyclones (Pearson's R = 0.92), Bayesian analysis approaches can overcome some of the reduction in the signal (Figures 3F and 3G), and 75% of genes identified by either of the screens are common to both (Figure 3H). Therefore, depending on the desired measurement precision, the cheaper and faster polyclonal screening will be sufficient for many purposes, and especially for identifying the strongest hits.

CRISPRi is as accurate as CRISPR in separating reference essential and non-essential genes

Next, we asked how the performance of the KRAB-MeCP2 CRISPRi construct compares with wild-type Cas9 screens. We previously conducted CRISPR-Cas9 screens in the same iPSC and K562 cell lines using a double gRNA library targeting 18,000 genes with 3 guides per gene (Peets et al., 2019). The CRISPRi screens compared favorably, with higher AUC values (0.98 for iPSC and 0.96 for K562) than CRISPR-Cas screens (0.95 for both lines; Figure 4A). The gene-level average gRNA $\log_2$ fold changes were moderately correlated between CRISPR and CRISPRi screens in the same cell line (Pearson's R = 0.42), which is in line with concordances of both knockout and repression screens in different types of stem cells (R = 0.37–0.53; Figure S4A). Furthermore, of the essential genes identified in one screen, about a half were also hits in the other (1,143/2,292 for CRISPR and 1,143/2,159 for CRISPRi; Figure 4F). Overall, both systems can identify essential genes, but differences in cell line context, growth conditions, the library used, screen coverage, and other experimental parameters generate substantial variation in performance and hit lists both within and between various screen types. CRISPRi screens can be cheaper in stem cells due to the lack of double-strand break toxicity, as well as give reasonable performance using polyclonal lines.

CRISPR and CRISPRi screens employ different mechanisms of action to perturb the target, which can bias the identified hits. For example, targeting the X chromosome with wtCas9 in lines from male donors creates DNA breaks

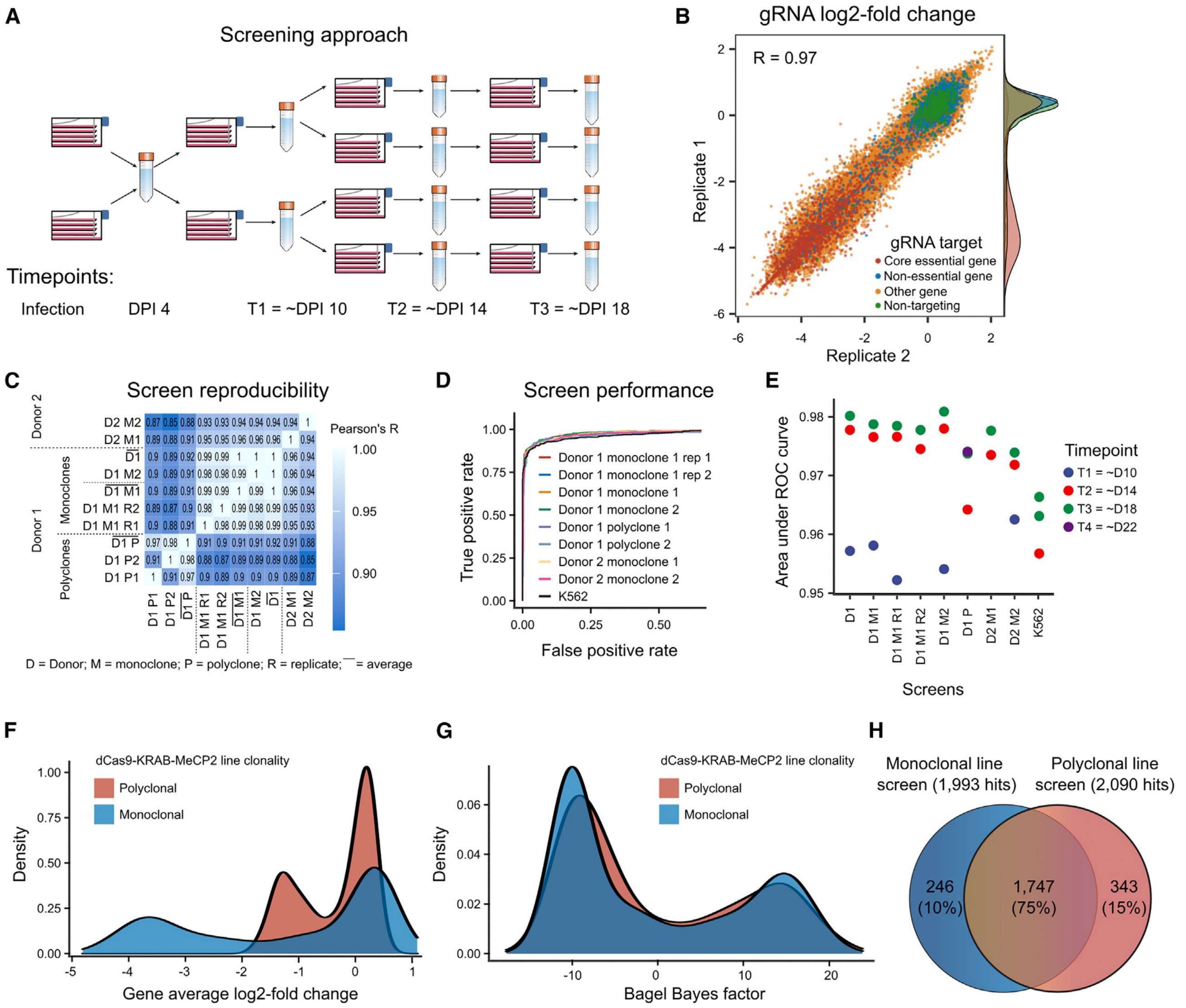

Figure 3. CRISPRi screening in iPSCs efficiently identifies essential genes

(A) Screening approach.

(B) Reproducibility of genome-wide screens in monoclonal iPSC lines. gRNA $\log_2$ fold change in replicate 1 (y axis) and replicate 2 (x axis). Red, essential genes; blue, non-essential genes; yellow, other genes; green, non-targeting controls.

(C) Concordance. Pearson's R of gene mean $\log_2$-scale gRNA fold changes (color) for screens performed in different donors, clonalities, clones, and replicates (x,y axis).

(D) Performance. True positive rate (y axis) at different false-positive rates (x axis) for separating gold standard essential from non-essential genes in different screens (colors).

(E) Time dependence. Area under the TPR-FPR curve (y axis) for different screens (x axis) across time (colors). Blue, T1 (about day 10); red, T2 (about day 14); green, T3 (about day 18); purple, T4 (about day 22).

(F) Density (y axis) of gene average $\log_2$ fold changes (x axis) of gold standard essential and non-essential genes as assayed in monoclonal (blue) and polyclonal (red) cell lines.

(G) As (F), but Bayes factor computed from BAGEL on the x axis.

(H) Venn diagram of hits found with monoclonal and polyclonal line screens.

on only one chromosome and is less toxic to cells. Thus, guides targeting single-copy chromosomes are enriched in CRISPR screens but not CRISPRi screens (Figure 4B), while non-specific guides are more depleted (Figure 4C). On the other hand, the wider "blast radius" of CRISPRi can cause false-positive results (Rosenbluh et al., 2017).

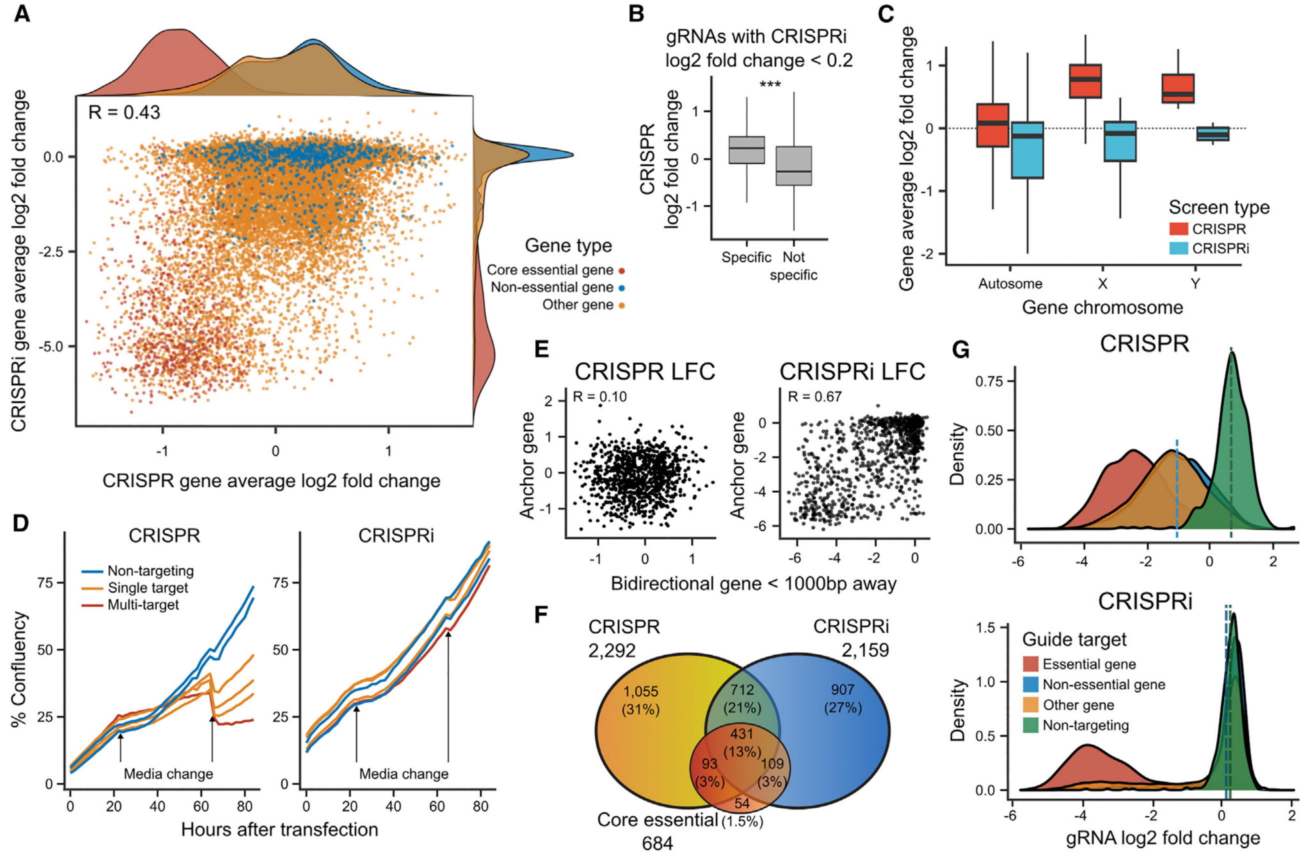

Figure 4. CRISPRi is as accurate as CRISPR in separating reference essential and non-essential genes

(A) Consistency of CRISPR and CRISPRi. gRNA $\log_2$ fold change in CRISPRi (y axis) and CRISPR screens (x axis). Red, essential genes; blue, non-essential genes; yellow, other genes; green, non-targeting controls.

(B) gRNA $\log_2$ fold change in CRISPR screen (y axis) for gRNAs that have no signal (absolute $\log_2$ fold change < 0.1) in CRISPRi screen, stratified to specific gRNAs with a single target (left), and non-specific ones (right). Box and whiskers as in Figure 2A. Wilcoxon test ***$p < 10^{-4}$

(C) Gene average $\log_2$ fold change (y axis) for genes on different types of chromosomes (x axis) for CRISPRi (blue) and CRISPR (red) screens. Box and whiskers as in Figure 2A.

(D) Percent confluency (y axis) across time since transfection (x axis) for gRNAs with different number of targets (colors). Blue, 0 targets; yellow, 1 target; red, 2 or more targets. Arrows, media change times.

(E) $\log_2$ fold change of gRNA (markers) at an anchor gene (x axis) and another gRNA at a bidirectional gene at most 1 kb away (y axis) for CRISPR screens (left panel) and CRISPRi screens (right panel).

(F) Venn diagram of overlap of CRISPR screen hits, CRISPRi screen hits, and gold standard essential genes.

(G) Density (y axis) of gRNA $\log_2$ fold change (x axis) for gRNAs in CRISPR screen (top panel) and CRISPRi screen (bottom panel). Red, essential gene targeting; blue, non-essential gene targeting; yellow, other gene targeting; green, non-targeting controls.

Indeed, when we compared nearby gene pairs with at most 1 kb distance between their TSSs, we observed a high correlation of signal in CRISPRi screens (R = 0.61) but not in CRISPR screens (Figure 4D), indicating a likely confounding due to targeting nearby TSSs.

The main drawback of CRISPR screens is double-strand break-induced cell death, which can be overcome by silencing *TP53* and p53 pathway genes (Haapaniemi et al., 2018). We first tested whether targeting *TP53* by CRISPRi in iPSCs also provides a growth advantage, and found it to confer a positive effect, but not as large as in CRISPR screens ($\log_2$ fold change upon *TP53* perturbation 1.04 by CRISPR vs. 0.55 by CRISPRi; Figure S4B). Next, we confirmed the deleterious effect of double-strand breaks in CRISPR but not CRISPRi screens. Indeed, the average depletion of non-targeting control gRNAs was close to that of gRNAs targeting non-essential genes for CRISPRi screen (0.2 vs. 0.1; Figure 4G), but not CRISPR screen (0.68 vs. −1.02). This implies that targeting alone with a wtCas9 construct carries a phenotypic impact in hiPSCs

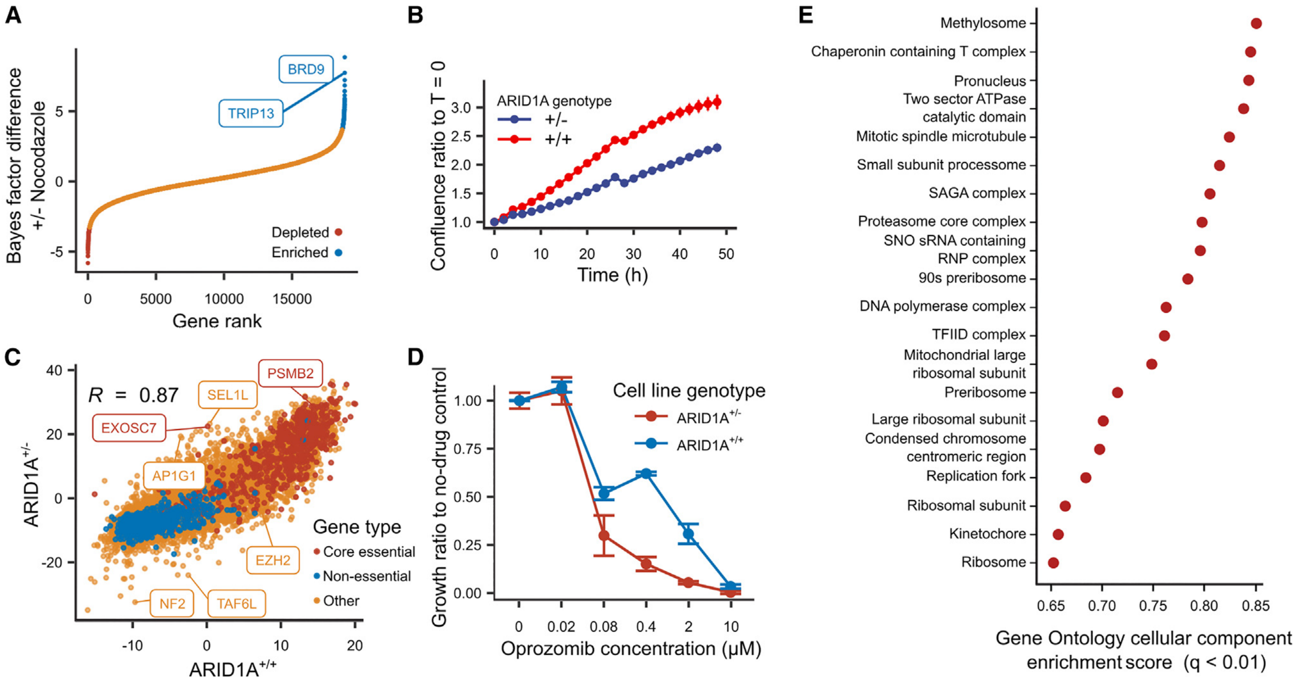

Figure 5. CRISPRi identifies context dependent essential genes

(A) BAGEL Bayes factor difference between screens with and without nocodazole treatment (y axis) in increasing value (x axis). Red, targeted genes significantly depleted in nocodazole; blue, targeted genes significantly enriched in nocodazole.

(B) Cell density relative to start time (y axis) across time (x axis) for iPSC lines with wild-type *ARID1A* genotype (red) and heterozygous loss of function (blue).

(C) BAGEL Bayes factor for genes (markers) for screens in *ARID1A* heterozygous knockout cell line (y axis) and no-mutation control (x axis). Red, essential genes; blue, non-essential genes; yellow, other genes.

(D) Growth relative to no drug control (y axis) at different concentrations of oprozimib (x axis) in $ARID1A^{+/+}$ (blue) and $ARID1A^{+/-}$ (red) cell lines. Mean ± standard error of the mean.

(E) Enrichment score (x axis) for different gene ontology cellular components (y axis).

(Aguirre et al., 2016; Haapaniemi et al., 2018). Finally, we validated that this effect is dose dependent, by comparing the growth of Cas9 and dCas9-KRAB-MeCP2 lines with guides that target zero, one, or multiple targets in the genome, and monitoring cell growth in a live imaging system. All three guides showed the same growth pattern in iPSC-dCas9-KRAB-MeCP2 lines, while the cutting at an additional number of targets caused increased cell death in iPSC-Cas9 cells (Figure 4D). Altogether, CRISPRi screens do not suffer from the dose-dependent double-strand break-induced cell death observed in CRISPR screens, but repressing the TP53 tumor suppressor still provides a moderate selective advantage.

CRISPRi identifies context-dependent essential genes

An important screening paradigm is identification of context-specific vulnerabilities for therapeutics and diagnostics. We therefore explored the utility of CRISPRi screens for mapping genome-scale drug-gene and allele-gene interactions. We first conducted pooled CRISPRi screens to identify genes that lead to nocodazole-dependent cell depletion (experimental procedures). Nocodazole is a well-established anti-mitotic agent that is less toxic in iPSCs. It arrests cells at G2/M phase by binding and destabilizing microtubules (Yiangou et al., 2019). The two genes most enriched in screens under nocodazole treatment compared with standard medium were *TRIP13* and *BRD9* (Figure 5A). *TRIP13* is a gene implicated in cell-cycle control mechanisms, and its depletion is known to slow cell division, but to also allow cells to escape nocodazole-induced cell-cycle arrest (Ma and Poon, 2016; Marks et al., 2017). *BRD9*, however, has a TSS only 80 bp apart from *TRIP13*, suggesting that the *BRD9* signal may be an unintended effect of the CRISPRi system and, indeed, *BRD9* was only a hit using CRISPRi (LFC < −3) but not CRISPR screening (LFC = 0.16). Thus, the nocodazole-dependent signal from *BRD9* is a likely false positive because of its TSS proximity to the known *TRIP13* causal

gene. Therefore, CRISPRi screening can identify gene-drug interactions, but the outputs need to be carefully analyzed to avoid modes of false positives not observed in CRISPR screens.

To identify mutation-specific gene effects, we screened for *ARID1A*-dependent vulnerabilities in iPSCs. The *ARID1A* gene produces a subunit of the ATP-dependent histone deacetylase SWI/SNF complex that has been implicated in developmental disorders, as well as 20% of all cancers (Pagliaroli and Trizzino, 2021) therefore potentially an important synthetic lethal gene dependency target for iPSCs. We produced isogenic iPSC lines by targeting the *ARID1A* coding region with transiently expressed Cas9, and picking one clonal line with a 7-nucleotide heterozygous deletion (c. 2389–2395: TCCAGCAGC>TC, p. 667–669: SSS/X), and one control line with wild-type sequence. The heterozygous mutation decreased *ARID1A* expression by 60% and resulted in slower growth compared with the wild-type line (Figure 5B). To prepare the cells for CRISPRi screening, we transfected the two lines with the dCas9-KRAB-MeCP2 construct and selected monoclonal cell lines with at least 65% silencing activity in the reporter assay. We proceeded with genome-wide screens in mutant and wild-type backgrounds using the Dolcetto library as described above. The depletion signals of the two screens were less correlated compared with replicates in wild-type cells (Pearson's R of BAGEL Bayes factors = 0.87 vs. 0.94; Figure 5C), suggesting that the *ARID1A* mutation altered the gene essentiality profile.

We next aimed to understand the differentially depletion-sensitive genes. Tumor suppressor gene *NF2* and chromatin remodeler gene *TAF6L* had the strongest increase of statistical signal in the mutant line (experimental procedures), while genes implicated in RNA and protein turnover (*EXOSC7* and *SEL1L*) and protein trafficking (*AP1G1*) had the most decrease. *SEL1L* encodes the adaptor subunit of ERAD ubiquitin ligase, which extracts misfolded proteins from the endoplasmic reticulum into the cytosol to be degraded by the proteasome (Hwang and Qi, 2018). Consistent with the role of protein degradation, inhibition of the proteasome 20S subunit beta 2 (*PSMB2*) gene, a potential drug target, resulted in more cell death in the $ARID1A^{+/-}$ line (Figure 5B). To confirm this differential growth effect of proteasome inhibition on *ARID1A* wild-type and mutant lines, we applied physiological concentrations of oprozomib, a second-generation proteasome inhibitor in trial for the treatment of hematological cancers (Sherman and Li, 2020), and measured the difference in growth rate. We found that *ARID1A* mutant line decreased in cell confluence more than wild-type cells upon oprozomib treatment when compared with the no drug control (Figure 5D). Our results thus confirm the sensitivity of *ARID1A* mutant cancer lines to proteotoxic stress (Tomihara et al., 2021), which could be therapeutically exploited.

More globally, gene set enrichment analysis of all the genes ranked by the difference between the wild-type and mutant line screen depletion identified the proteasome core complex among the top 20 enriched pathways (Figure 5E), as well as other pathways related to *ARID1A* function, like centromere complex assembly and kinetochore organization, confirming previous results (Caumanns et al., 2018; Mathur, 2018). Furthermore, a String database (Szklarczyk et al., 2021) analysis with 200 most depleted genes identified three clusters: gene expression regulation, cell-cycle regulation, and cholesterol biosynthesis regulation (Figure S5) around ARID1A and two other members of SWI/SNF family (SS18 and SMARCE1). We also tested whether essentiality changes upon ARID1A dose reduction mimic those in cancer cell lines upon damaging mutations to the ARID1A gene, but found no strong signal (Note S1).

Context-dependent vulnerabilities replicate with different reagents and assays

We confirmed the genotype- and small-molecule-dependent fitness effects with alternative reagents and assays. First, to validate that the reduced dose of *ARID1A*, rather than any other clonal mutation, leads to growth rate reduction in the *ARID1A* mutant line, we tested six gRNAs targeting 17, 59, 894, 987, 1,160, and 1,314 nt downstream of the TSS of *ARID1A* in wild-type iPSCs (experimental procedures). Two of the gRNAs, targeting 17 and 1,314 nt downstream of TSS, caused substantial growth retardation in the first 36 h after transfection (Figure 6A).

Second, we confirmed the impact of silencing *EZH2*, coding for the catalytic domain of histone methyltransferase polycomb repressive complex 2 (Figure 5B), which was more essential in the wild-type screen compared with the one in the *ARID1A* mutant line. We transfected cells with either *EZH2* targeting or non-targeting gRNAs and, after 9 days in culture, observed substantial morphology changes upon targeting compared with non-targeting guides in $ARID1A^{+/+}$ cells and *EZH2*-silenced $ARID1A^{+/-}$ cells (Figure S6A). Furthermore, 32% of *EZH2*-silenced $ARID1A^{+/+}$ cells were negative for the stem cell marker hTRA-1-60 (Figure 6B), indicating likely differentiation from stem cell state toward a less propagating lineage with different morphology and expression profile, and implicating *ARID1A* as a causal intermediate factor in this process.

Third, we validated hits from the whole-genome screens. We compared the signal between CRISPRi screens in $ARID1A^{+/+}$ and $ARID1A^{+/-}$ lines and picked 1,657 genes with large differences (experimental procedures) to re-measure in a follow-up experiment. Screens in the wild-type

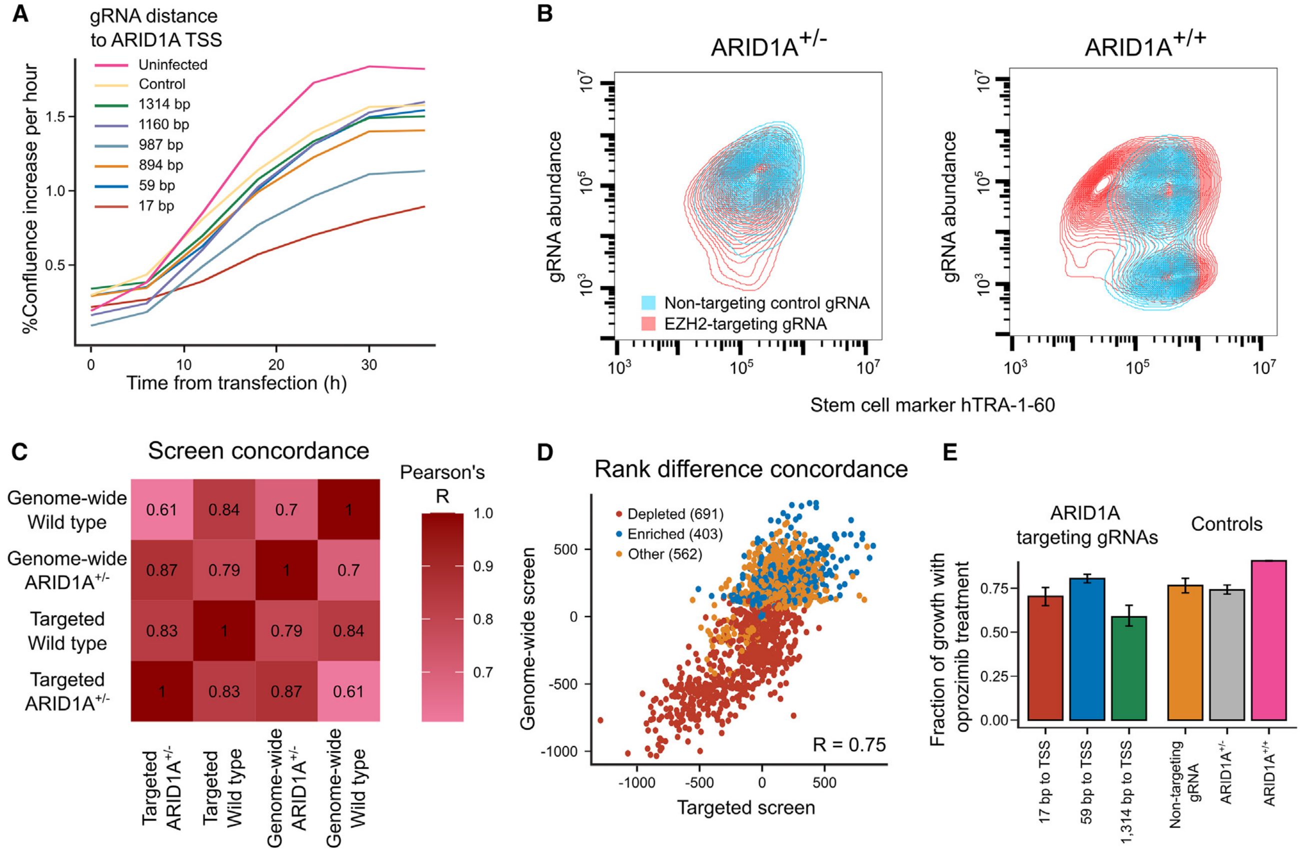

Figure 6. Context-dependent vulnerabilities replicate with different reagents and assays

(A) Percent confluence increase per hour (y axis) across time from transfection (x axis) of guide RNAs targeting different distances to the ARID1A gene TSS (colors) into iPSCs.

(B) Density curves of stem cell marker hTRA-1-60 signal (x axis) for different gRNA plasmid abundance proxies (y axis) in ARID1A$^{+/-}$ (left panel) and ARID1A$^{+/+}$ (right panel) cell lines. Red, EZH2-targeting gRNA; blue, non-targeting control gRNA.

(C) Screen concordance (Pearson's R of gene average $\log_2$ fold changes; colors) of genome-wide and targeted CRISPRi screens in ARID1A$^{+/+}$ and ARID1A$^{+/-}$ cells (x and y axes).

(D) Rank difference of genes (markers) between CRISPRi screens in ARID1A$^{+/+}$ and ARID1A$^{+/-}$ cells in a genome-wide screen (y axis) and targeted follow-up screen (x axis). Red, depleted genes in the genome-wide screen; blue, enriched genes; orange, other genes.

(E) Fraction of growth upon oprozomib treatment compared with no drug control (y axis) for ARID1A targeting gRNAs (x axis; first three bars) and controls (last three bars). mean± standard error of the mean.

and mutant lines using a gRNA library against these genes were consistent with the original ones both in absolute terms (Pearson's R of gene LFCs > 0.84; Figures 6C and S6B), as well as for difference in the mutant line (Pearson's R of rank changes 0.75; Figure 6D).

Finally, to confirm that sensitivity to proteasome inhibition is due to *ARID1A* gene targeting, we treated the ARID1A wild-type lines with the two strongly growth-retarding *ARID1A*-targeting gRNAs, one weak gRNA, a negative control gRNA, and no gRNA, as well as with oprozomib (1 μg/mL) for 1 day and compared growth with non-treated controls, and the *ARID1A*$^{+/-}$ line. The vulnerability of hiPSCs to oprozomib treatment was dependent on *ARID1A* targeting, with gRNAs leading to stronger growth effect in hiPSCs also giving rise to stronger phenotypes upon proteasome inhibition, resembling the growth of the mutant line (Figure 6E). Thus, we have demonstrated that an important developmental disorder and cancer gene can potentially be therapeutically targeted with a small molecule in a dose-dependent manner.

DISCUSSION

We presented the first systematic investigation of genome-wide CRISPRi screens in hiPSCs and demonstrated

that CRISPRi is an efficient and safe alternative to standard CRISPR-Cas-based screening. We identified dCas9-KRAB-MeCP2 as the most potent fusion protein for target silencing in hiPSCs, with a range that can extend to 1.4 kb into the coding region. Whole-genome CRISPRi screens using this construct performed as well as different novel and previously published screens with standard Cas9. We identified actionable drug sensitivity for *ARID1A* mutant iPSCs, and other interactions illuminating the role of *ARID1A* for stem cell growth and differentiation.

The best silencing modality is not easy to choose for a new experiment. For example, dCas9-KRAB is effective when integrated into the AAVS1 locus in hiPSCs (Mandegar et al., 2016), but replicating this setup is cumbersome for screening in multiple lines. The dCas9-KRAB-MeCP2 performed even better for silencing (Yeo et al., 2018), but varied in efficiency across cell types. By testing three constructs with different delivery methods and both monoclonal and polyclonal versions, we found that monoclonal lines expressing the dCas9-KRAB-MeCP2 fusion protein showed both best silencing activity with the reporter construct and best performance in genome-wide screens.

Another important consideration in practice is the cost required for screening at scale. Both standard CRISPR and CRISPRi screens can identify essential genes in an experiment (Gilbert et al., 2014; Horlbeck et al., 2016; Sanson et al., 2018), and while CRISPR has previously been argued to be more sensitive (Rosenbluh et al., 2017; Sanson et al., 2018), CRISPRi outperformed CRISPR in both hiPSC and K562 cells in our hands, at similar screen coverages and screen durations. In particular, we observed that a 14-day duplicate screen with 50× coverage at infection and 200× during passaging gives near-optimal results, greatly reducing the cost per screen compared with more standard larger designs. Efficacy, resolution, budget, and labor for a screen can be optimized by choosing mono- or polyclonal lines, screen coverage, and duration.

The dCas9 protein has to be targeted near the TSS for efficient screening (Gilbert et al., 2013), but the blast radius varies for different fusion proteins. The best window for dCas9-KRAB activity has been established to be 0 to 100 bp after the TSS (Radzisheuskaya et al., 2016; Rosenbluh et al., 2017; Sanson et al., 2018; Yeo et al., 2018). However, these reports are specific to the KRAB fusion and explored only a few target sites. We demonstrated that the target range of dCas9-KRAB-MeCP2 in iPSCs is larger, with impacts on gene expression as far as 1.4 kb after the TSS, and with the efficiency gradually decreasing with distance. We propose that this understanding could be used as a way to modulate the extent of downregulation, as has been previously done with mismatched gRNAs (Jost et al., 2020). This fine-grained control would be useful to study haploinsufficiency of target genes, and other questions that require a more precise range of the gene dose. The large impact range of dCas9-KRAB-MeCP2 also decreases the false-negative rate due to alternative TSSs for different cell types. We showed that false-negative results rarely occur for the majority of genes for which the alternative is less than 1 kb away.

CRISPR screens have already yielded gene-gene interactions (Horlbeck et al., 2018; Kim et al., 2022). We screened for interactions with the *ARID1A* gene, implicated in both developmental disorders (OMIM: 135900) as well as cancer (Pagliaroli and Trizzino, 2021), suggesting growth control to be an important aspect of its function. Suppressing the *NF2* gene in the *ARID1A* mutant background gave iPSCs a growth advantage and, although direct interaction of *ARID1A* and *NF2* is not known, both proteins regulate pathways inhibiting oncogenic YAP/TAZ genes, and their double mutation causes hepatocellular carcinoma in mice (Chang et al., 2018; Patel et al., 2017). Two other hits, *TAF6L* and *EZH2*, are members of the SAGA and PRC2 chromatin-modifying complexes, interacting with SWI/SNF in cell fate determination (Pagliaroli and Trizzino, 2021; Seruggia et al., 2019). Further investigation of the *EZH2* effect with an arrayed screen demonstrated the growth advantage in the *ARID1A*$^{+/-}$ background was due to inhibition of cell differentiation, which further illuminates *ARID1A* function in cell fate determination.

Pluripotent stem cells of different provenance and capacity are poised to expand our understanding and ability to engineer disease mutations, cell types, entire organs, and courses of development (Hanna et al., 2002; Liu et al., 2020). Precise methods for genome engineering to enable control in these systems is a crucial enabling technology for this progress. Detection of context-specific effects will be key both for accurately targeting only the diseased state, as well as to chart the inherent heterogeneity that needs to be accounted for.

EXPERIMENTAL PROCEDURES

Resource availability

Corresponding authors

Further inquiries and requests for data and resources should be directed to corresponding authors, Sunay Usluer (sunay.usluer@sanger.ac.uk) and Leopold Parts (leopold.parts@sanger.ac.uk).

Materials availability

Libraries and plasmids uniquely produced through this study will be made available upon request and will require a material transfer agreement.

Data and code availability

Data and analyses for this study are available from the following git hub repository: https://github.com/sunayusluer/CRISPRi-Analysis.

Cell culture

K562-Cas9 and K562-Cas9-GFP cells were cultured in RPMI supplemented with 10% FCS, 2 mM L-glutamine, 100 U/mL penicillin, and 100 mg/mL streptomycin (will be referred to as supplemented RPMI medium hereafter). Cells were passaged 1:20 every 4 days. Wild-type hiPSC (fiaj-1 and kolf2) lines were obtained from the HipSci platform, where its karyotype and phenotype were evaluated to be normal. hiPSC lines were cultured on Vitronectin-XF (STEMCELL Technologies, 07180)-coated plates and in mTeSR-Plus Media (STEMCELL Technologies, 100–0276). For maintenance, they were passaged every 4 days as clumps using ReLeSR (STEMCELL Technologies, 05872) according to the producer's protocol. All cell lines were cultured at 37°C, 5% CO_2.

Cloning stable dCas9 fusion protein-expressing lines

Three different dCas9 fusion proteins were tested for dCas9 activity in both K562 and hiPSC lines: pLX_311-KRAB-dCas9 (Addgene 96918), pB-CAGGS-dCas9-KRAB-MeCP2 (Addgene, 110824), and pPB-dCas9-KRAB (kindly provided by Dr. Qianxin Wu). The first is a lentiviral delivery vector, whereas the latter two rely on the piggyBac transposase for genome integration. We produced hiPSC and K562 lines stably expressing all three fusion proteins by transducing/transfecting them with the above vectors (supplemental experimental procedures).

Producing monoclonal hiPSC lines

After 10 days of blasticidin selection, polyclonal cell populations were collected with accutase and 1,000 cells seeded into 6-cm dishes precoated with Synthemax II-SC Substrate (Corning, 3535) at a concentration of 5 μg/cm^2 in mTeSR-E8 medium supplemented with 10× CloneR (Stem Cells, 05888). The medium was changed every day with blasticidin-supplemented mTeSR+Plus medium (10 μg/m). After 10 days, visible colonies were manually transferred into 12-well plates precoated with Vitronectin XF. Surviving monoclonal cell lines were expanded and used in later analyses.

Producing monoclonal K562 lines

After 10 days of blasticidin selection, monoclonal lines were produced by serial dilution as follows: polyclonal cells were counted and serial diluted to an estimated 50 cells in 20 mL K562 medium and 200 μL of suspension was distributed into each well of a 96-well plate. In the first iteration, there was cell growth in 6 wells. Cells from these 6 wells were separately collected, diluted, and redistributed into 6 different 96-well plates, and one well from each plate was picked as monoclonal lines.

Testing CRISPRi efficacy

Monoclonal and polyclonal hiPSC-dCas9 lines were transfected with pCS2-iREP-GFP-PGK-BFP-U6-gRNA-irep or pCS2-iREP-GFP-PGK-BFP-U6-gRNA-mock vectors (cloning details are in supplemental experimental procedures) as replicates in 12-well plates using TransIT-LT1 Transfection Reagent (Mirus Bio, MIR2300) according to the manufacturer's instructions. Similarly, monoclonal and polyclonal K562-dCas9 lines were transfected with pCS2-iREP-GFP-PGK-BFP-U6-gRNA-irep or pCS2-iREP-GFP-PGK-BFP-U6-gRNA-mock vectors in replicate with Lipofectamine LTX reagent, with non-fluorescent plasmid as negative control. Three days after transfection, hiPSCs were harvested with accutase and K562 cells were collected by centrifugation. All cells were washed and resuspended in PBS+FBS (2%). Harvested cells were analyzed in a CytoFLEX flow cytometer using FlowJo analysis software (Beckman Coulter). CRISPRi efficacy was quantified as percent activity – the percent decrease in median GFP level in BFP-positive cells.

Generating the CRISPRi tiling library

Design

To determine the optimal target window of dCas9-KRAB-MeCP2 in iPSCs, three sub-libraries were designed (Figure S2A). First, the TSS tiling library placed guides relative to the TSSs in hiPSCs, as identified from the CAGE annotations in the Fantom database (Lizio et al., 2015) according to the GRCh38 reference genome. Within the TSS tiling library, the "TSS Tiling, Extra" library tiled from 0 to +100 bp of TSS site for the single transcript of 882 genes essential in hiPSCs (Figure 2A); the "TSS Tiling, Different" library tiled from −200 to +300 bp of the TSS site for the top 2 transcripts of 20 essential genes for which the TSS annotation in hiPSCs did not match the canonical one (Figure 2B); and the "TSS Tiling, Other" library tiled the top 2 transcripts of 20 essential genes. Second, the Gene tiling library targeted guides to all PAMs in coding sequence of a single transcript of 451 Hart essential genes, and 36 non-essential genes (Figure 2C). Finally, 200 non-targeting guides were used as control (Table S1).

The designed library was produced as complex oligonucleotide pools (Genescript) and cloned into lentiviral gRNA expression vector pKLV2-U6gRNA5(BbsI)-ccdb-PGKpuroBFP-W (AddGene: 67974) (Tzelepis et al., 2016) (supplemental experimental procedures).

gRNAs for iPSC-specific alternative TSSs for 10 genes were designed using the CRISPick tool of the Broad Institute (Doench et al., 2016; Sanson et al., 2018).

CRISPRi screening

The Dolcetto library (Sanson et al., 2018) was acquired from Addgene (no. 1000000114) as two plasmid pools, each with a half-library (sets A and B) in the XPR_500 backbone. Only the Dolcetto set A plasmid pool was used in this study. Plasmid pools were re-amplified, packed into lentivirus, and titrated as detailed in supplemental experimental procedures.

hiPSCs

Cells (6 × 10^7) were mixed with the lentiviral Dolcetto library to achieve an estimated 0.3 MOI and seeded on 2× vitronectin-coated 5-layer flasks at 36K cells/cm^2 density in mTeSR-Plus medium with Rock inhibitor (Ri) (10 μM, Y-27632, STEMCELL Technologies, 72304). The next day, the medium was changed to remove Ri. On day 3 post infection, puromycin selection was started at 0.5 μg/mL, and continued through the screen duration (17–22 days). Cells were passaged at 90% confluency and seeded back at 16K cells/cm^2 density for the rest of the screen. The remaining cell pellets were aliquoted and frozen for genomic DNA extraction.

K562 cells

Cells (75 × 10^6) were resuspended in supplemented RPMI+ polybrene (8 μg/mL) medium and mixed with the Dolcetto library virus aiming for a MOI of 0.3. The cell + virus suspension was centrifuged at 1,000 rcf for 30 min. The cells were then resuspended in the same medium and plated into two T150cm^2 plates for a final

concentration of 0.45 × 10^6 cells/mL. The infected cells were selected with puromycin (2 μg/mL) starting from day 1 after infection until day 8. After complete selection, the cells in culture were passaged every 3 or 4 days, and plated back at 0.1 × 10^6 cells/mL density. Aliquoted pellets of at least 40 × 10^6 cells were frozen for genomic DNA extraction.

Genomic DNA isolation and sequencing library preparation were conducted as reported previously (Peets et al., 2019) and as in supplemental experimental procedures.

Data analysis

gRNA sequences were counted from fasta files and aligned to corresponding library maps without allowing any mismatches. Uniformity of plasmid libraries was determined using the Gini index as implemented by Wang et al. (2019). Average sequencing coverage of all libraries was around 400×. When all technical replicates were combined together, a single screen had around 2,000× coverage for final time points. Raw counts in each sequencing sample were normalized and transformed to $\log_2$ scale according to the formula: $\log_2(((\text{reads per guide}/\text{sum of all read counts}) \times 10^6) + 1)$. Log_2 fold change per guide was calculated by subtracting $\log_2$ transformed and normalized read counts of final time points from plasmid library sequences. To determine essential and non-essential genes, Bagel v.2 (Kim and Hart, 2021) was used with default parameters. To find differentially essential genes between $ARID1A^{+/+}$ and $ARID1A^{+/-}$ lines, we calculated the mean and standard deviation of the difference across all genes, and assigned the genes with values of at least two standard deviations away from the mean to be differentially essential.

SUPPLEMENTAL INFORMATION

Supplemental information can be found online at https://doi.org/10.1016/j.stemcr.2023.03.008.

AUTHOR CONTRIBUTIONS

S.U. and L.P. conceptualized and initiated the study with the help of L.C. S.U. performed experiments with the help of Y.Z., K.U., and C.D. S.U. and P.H. analyzed the data with the help of J.S., G.N., and O.E.G. K.A. designed the TSS tiling library. B.N. created isogenic hiPSC lines with the supervision of S.S.G. and O.M.D. L.P. supervised the project. S.U. and L.P. wrote the manuscript with input from all authors.

ACKNOWLEDGMENTS

S.U. was supported by the Janet Thornton Fellowship. P.H., L.C., Y.Z., K.U., C.D., J.S., G.N., O.E.G., S.G., B.N., O.M.D., and L.P. were supported by Wellcome (grant nos. 108413/A/15/D and 220540/Z/20/A, “Wellcome Sanger Institute Quinquennial Review 2021–2026”). K.A. was supported by the Estonian Research Council (PSG415) and the Archimedes Foundation (EXCITE TK148).

The authors acknowledge the Wellcome Trust Sanger Institute as the source of (fiaj-1 and kolf-2) human induced pluripotent cell lines, which were generated under the Human Induced Pluripotent Stem Cell Initiative funded by a grant from the Wellcome Trust and the Medical Research Council, supported by the Wellcome Trust (WT098051) and the NIHR/Wellcome Trust Clinical Research Facility, and also acknowledge the Life Science Technologies Corporation as set out at Schedule 1.

CONFLICT OF INTERESTS

L.C. and O.M.D. receive remuneration and stock options from bit.bio.

Received: September 17, 2022
Revised: March 7, 2023
Accepted: March 13, 2023
Published: April 6, 2023

REFERENCES

Aguirre, A.J., Meyers, R.M., Weir, B.A., Vazquez, F., Zhang, C.-Z., Ben-David, U., Cook, A., Ha, G., Harrington, W.F., Doshi, M.B., et al. (2016). Genomic copy number dictates a gene-independent cell response to CRISPR/Cas9 targeting. Cancer Discov. *6*, 914–929.

Alerasool, N., Segal, D., Lee, H., and Taipale, M. (2020). An efficient KRAB domain for CRISPRi applications in human cells. Nat. Methods *17*, 1093–1096.

Behan, F.M., Iorio, F., Picco, G., Gonçalves, E., Beaver, C.M., Migliardi, G., Santos, R., Rao, Y., Sassi, F., Pinnelli, M., et al. (2019). Prioritization of cancer therapeutic targets using CRISPR–Cas9 screens. Nature *568*, 511–516.

Caumanns, J.J., Wisman, G.B.A., Berns, K., van der Zee, A.G.J., and de Jong, S. (2018). ARID1A mutant ovarian clear cell carcinoma: a clear target for synthetic lethal strategies. Biochim. Biophys. Acta. Rev. Cancer *1870*, 176–184.

Chang, L., Azzolin, L., Di Biagio, D., Zanconato, F., Battilana, G., Lucon Xiccato, R., Aragona, M., Giulitti, S., Panciera, T., Gandin, A., et al. (2018). The SWI/SNF complex is a mechanoregulated inhibitor of YAP and TAZ. Nature *563*, 265–269.

Doench, J.G., Fusi, N., Sullender, M., Hegde, M., Vaimberg, E.W., Donovan, K.F., Smith, I., Tothova, Z., Wilen, C., Orchard, R., et al. (2016). Optimized sgRNA design to maximize activity and minimize off-target effects of CRISPR-Cas9. Nat. Biotechnol. *34*, 184–191.

Evers, B., Jastrzebski, K., Heijmans, J.P.M., Grernrum, W., Beijersbergen, R.L., and Bernards, R. (2016). CRISPR knockout screening outperforms shRNA and CRISPRi in identifying essential genes. Nat. Biotechnol. *34*, 631–633.

Funk, L., Su, K.-C., Ly, J., Feldman, D., Singh, A., Moodie, B., Blainey, P.C., and Cheeseman, I.M. (2022). The phenotypic landscape of essential human genes. Cell *185*, 4634–4653.e22.

Gilbert, L.A., Larson, M.H., Morsut, L., Liu, Z., Brar, G.A., Torres, S.E., Stern-Ginossar, N., Brandman, O., Whitehead, E.H., Doudna, J.A., et al. (2013). CRISPR-mediated modular RNA-guided regulation of transcription in eukaryotes. Cell *154*, 442–451.

Gilbert, L.A., Horlbeck, M.A., Adamson, B., Villalta, J.E., Chen, Y., Whitehead, E.H., Guimaraes, C., Panning, B., Ploegh, H.L., Bassik, M.C., et al. (2014). Genome-scale CRISPR-mediated control of gene repression and activation. Cell *159*, 647–661.

Haapaniemi, E., Botla, S., Persson, J., Schmierer, B., and Taipale, J. (2018). CRISPR–Cas9 genome editing induces a p53-mediated DNA damage response. Nat. Med. *24*, 927–930.

Hanna, R.E., and Doench, J.G. (2020). Design and analysis of CRISPR-Cas experiments. Nat. Biotechnol. *38*, 813–823.

Hanna, L.A., Foreman, R.K., Tarasenko, I.A., Kessler, D.S., and Labosky, P.A. (2002). Requirement for Foxd3 in maintaining pluripotent cells of the early mouse embryo. Genes Dev. *16*, 2650–2661.

Hart, T., Brown, K.R., Sircoulomb, F., Rottapel, R., and Moffat, J. (2014). Measuring error rates in genomic perturbation screens: gold standards for human functional genomics. Mol. Syst. Biol. *10*, 733.

Hart, T., Chandrashekhar, M., Aregger, M., Steinhart, Z., Brown, K.R., MacLeod, G., Mis, M., Zimmermann, M., Fradet-Turcotte, A., Sun, S., et al. (2015). High-resolution CRISPR screens reveal fitness genes and genotype-specific cancer liabilities. Cell *163*, 1515–1526.

Hart, T., Tong, A.H.Y., Chan, K., Van Leeuwen, J., Seetharaman, A., Aregger, M., Chandrashekhar, M., Hustedt, N., Seth, S., Noonan, A., et al. (2017). Evaluation and design of genome-wide CRISPR/SpCas9 knockout screens. G3 *7*, 2719–2727.

Horlbeck, M.A., Gilbert, L.A., Villalta, J.E., Adamson, B., Pak, R.A., Chen, Y., Fields, A.P., Park, C.Y., Corn, J.E., Kampmann, M., and Weissman, J.S. (2016). Compact and highly active next-generation libraries for CRISPR-mediated gene repression and activation. Elife *5*, e19760. https://doi.org/10.7554/eLife.19760.

Horlbeck, M.A., Xu, A., Wang, M., Bennett, N.K., Park, C.Y., Bogdanoff, D., Adamson, B., Chow, E.D., Kampmann, M., Peterson, T.R., et al. (2018). Mapping the genetic landscape of human cells. Cell *174*, 953–967.e22.

Hwang, J., and Qi, L. (2018). Quality control in the endoplasmic reticulum: crosstalk between ERAD and UPR pathways. Trends Biochem. Sci. *43*, 593–605.

Ihry, R.J., Worringer, K.A., Salick, M.R., Frias, E., Ho, D., Theriault, K., Kommineni, S., Chen, J., Sondey, M., Ye, C., et al. (2018). p53 inhibits CRISPR–Cas9 engineering in human pluripotent stem cells. Nat. Med. *24*, 939–946.

Ihry, R.J., Salick, M.R., Ho, D.J., Sondey, M., Kommineni, S., Paula, S., Raymond, J., Henry, B., Frias, E., Wang, Q., et al. (2019). Genome-scale CRISPR screens identify human pluripotency-specific genes. Cell Rep. *27*, 616–630.e6.

Jost, M., Santos, D.A., Saunders, R.A., Horlbeck, M.A., Hawkins, J.S., Scaria, S.M., Norman, T.M., Hussmann, J.A., Liem, C.R., Gross, C.A., and Weissman, J.S. (2020). Titrating gene expression using libraries of systematically attenuated CRISPR guide RNAs. Nat. Biotechnol. *38*, 355–364.

Kim, E., and Hart, T. (2021). Improved analysis of CRISPR fitness screens and reduced off-target effects with the BAGEL2 gene essentiality classifier. Genome Med. *13*, 2.

Kim, E., Novak, L.C., Lin, C., Colic, M., Bertolet, L.L., Gheorghe, V., Bristow, C.A., and Hart, T. (2022). Dynamic rewiring of biological activity across genotype and lineage revealed by context-dependent functional interactions. Genome Biol. *23*, 140.

Liu, G., David, B.T., Trawczynski, M., and Fessler, R.G. (2020). Advances in pluripotent stem cells: history, mechanisms, technologies, and applications. Stem Cell Rev. Rep. *16*, 3–32.

Lizio, M., Harshbarger, J., Shimoji, H., Severin, J., Kasukawa, T., Sahin, S., Abugessaisa, I., Fukuda, S., Hori, F., Ishikawa-Kato, S., et al. (2015). Gateways to the FANTOM5 promoter level mammalian expression atlas. Genome Biol. *16*, 22.

Ma, H.T., and Poon, R.Y.C. (2016). TRIP13 regulates both the activation and inactivation of the spindle-assembly checkpoint. Cell Rep. *14*, 1086–1099.

Mair, B., Tomic, J., Masud, S.N., Tonge, P., Weiss, A., Usaj, M., Tong, A.H.Y., Kwan, J.J., Brown, K.R., Titus, E., et al. (2019). Essential gene profiles for human pluripotent stem cells identify uncharacterized genes and substrate dependencies. Cell Rep. *27*, 599–615.e12. https://doi.org/10.1016/j.celrep.2019.02.041.

Mali, P., Yang, L., Esvelt, K.M., Aach, J., Guell, M., DiCarlo, J.E., Norville, J.E., and Church, G.M. (2013). RNA-guided human genome engineering via Cas9. Science *339*, 823–826.

Mandegar, M.A., Huebsch, N., Frolov, E.B., Shin, E., Truong, A., Olvera, M.P., Chan, A.H., Miyaoka, Y., Holmes, K., Spencer, C.I., et al. (2016). CRISPR interference efficiently induces specific and reversible gene silencing in human iPSCs. Cell Stem Cell *18*, 541–553.

Marks, D.H., Thomas, R., Chin, Y., Shah, R., Khoo, C., and Benezra, R. (2017). Mad2 overexpression uncovers a critical role for TRIP13 in mitotic exit. Cell Rep. *19*, 1832–1845.

Mathur, R. (2018). ARID1A loss in cancer: towards a mechanistic understanding. Pharmacol. Ther. *190*, 15–23.

Miles, L.A., Garippa, R.J., and Poirier, J.T. (2016). Design, execution, and analysis of pooled in vitro CRISPR/Cas9 screens. FEBS J. *283*, 3170–3180.

Pagliaroli, L., and Trizzino, M. (2021). The evolutionary conserved SWI/SNF subunits ARID1A and ARID1B are key modulators of pluripotency and cell-fate determination. Front. Cell Dev. Biol. *9*, 643361.

Patel, S.H., Camargo, F.D., and Yimlamai, D. (2017). Hippo signaling in the liver regulates organ size, cell fate, and carcinogenesis. Gastroenterology *152*, 533–545.

Peets, E.M., Crepaldi, L., Zhou, Y., Allen, F., Elmentaite, R., Noell, G., Turner, G., Iyer, V., and Parts, L. (2019). Minimized double guide RNA libraries enable scale-limited CRISPR/Cas9 screens. Preprint at bioRxiv. https://doi.org/10.1101/859652.

Przybyla, L., and Gilbert, L.A. (2022). A new era in functional genomics screens. Nat. Rev. Genet. *23*, 89–103.

Qi, L.S., Larson, M.H., Gilbert, L.A., Doudna, J.A., Weissman, J.S., Arkin, A.P., and Lim, W.A. (2013). Repurposing CRISPR as an RNA-guided platform for sequence-specific control of gene expression. Cell *152*, 1173–1183. https://doi.org/10.1016/j.cell.2013.02.022.

Radzisheuskaya, A., Shlyueva, D., Müller, I., and Helin, K. (2016). Optimizing sgRNA position markedly improves the efficiency of CRISPR/dCas9-mediated transcriptional repression. Nucleic Acids Res. *44*, e141.

Rosenbluh, J., Xu, H., Harrington, W., Gill, S., Wang, X., Vazquez, F., Root, D.E., Tsherniak, A., and Hahn, W.C. (2017).

Complementary information derived from CRISPR Cas9 mediated gene deletion and suppression. Nat. Commun. *8*, 15403–15408.

Sanson, K.R., Hanna, R.E., Hegde, M., Donovan, K.F., Strand, C., Sullender, M.E., Vaimberg, E.W., Goodale, A., Root, D.E., Piccioni, F., and Doench, J.G. (2018). Optimized libraries for CRISPR-Cas9 genetic screens with multiple modalities. Nat. Commun. *9*, 5416. https://doi.org/10.1038/s41467-018-07901-8.

Seruggia, D., Oti, M., Tripathi, P., Canver, M.C., LeBlanc, L., Di Giammartino, D.C., Bullen, M.J., Nefzger, C.M., Sun, Y.B.Y., Farouni, R., et al. (2019). TAF5L and TAF6L maintain self-renewal of embryonic stem cells via the MYC regulatory network. Mol. Cell *74*, 1148–1163.e7.

Shalem, O., Sanjana, N.E., Hartenian, E., Shi, X., Scott, D.A., Mikkelson, T., Heckl, D., Ebert, B.L., Root, D.E., Doench, J.G., and Zhang, F. (2014). Genome-scale CRISPR-Cas9 knockout screening in human cells. Science *343*, 84–87.

Sherman, D.J., and Li, J. (2020). Proteasome inhibitors: harnessing proteostasis to combat disease. Molecules *25*, 671. https://doi.org/10.3390/molecules25030671.

Szklarczyk, D., Gable, A.L., Nastou, K.C., Lyon, D., Kirsch, R., Pyysalo, S., Doncheva, N.T., Legeay, M., Fang, T., Bork, P., et al. (2021). The STRING database in 2021: customizable protein-protein networks, and functional characterization of user-uploaded gene/measurement sets. Nucleic Acids Res. *49*, D605–D612.

Tian, R., Gachechiladze, M.A., Ludwig, C.H., Laurie, M.T., Hong, J.Y., Nathaniel, D., Prabhu, A.V., Fernandopulle, M.S., Patel, R., Abshari, M., et al. (2019). CRISPR interference-based platform for multimodal genetic screens in human iPSC-derived neurons. Neuron *104*, 239–255.e12.

Tomihara, H., Carbone, F., Perelli, L., Huang, J.K., Soeung, M., Rose, J.L., Robinson, F.S., Lissanu Deribe, Y., Feng, N., Takeda, M., et al. (2021). Loss of ARID1A promotes epithelial–mesenchymal transition and sensitizes pancreatic tumors to proteotoxic stress. Cancer Res. *81*, 332–343.

Tzelepis, K., Koike-Yusa, H., De Braekeleer, E., Li, Y., Metzakopian, E., Dovey, O.M., Mupo, A., Grinkevich, V., Li, M., Mazan, M., et al. (2016). A CRISPR dropout screen identifies genetic vulnerabilities and therapeutic targets in acute myeloid leukemia. Cell Rep. *17*, 1193–1205.

Wang, B., Wang, M., Zhang, W., Xiao, T., Chen, C.-H., Wu, A., Wu, F., Traugh, N., Wang, X., Li, Z., et al. (2019). Integrative analysis of pooled CRISPR genetic screens using MAGeCKFlute. Nat. Protoc. *14*, 756–780.

Wang, T., Wei, J.J., Sabatini, D.M., and Lander, E.S. (2014). Genetic screens in human cells using the CRISPR-Cas9 system. Science *343*, 80–84.

Wang, T., Pine, A.R., Kotini, A.G., Yuan, H., Zamparo, L., Starczynowski, D.T., Leslie, C., and Papapetrou, E.P. (2021). Sequential CRISPR gene editing in human iPSCs charts the clonal evolution of myeloid leukemia and identifies early disease targets. Cell Stem Cell *28*, 1074–1089.e7. https://doi.org/10.1016/j.stem.2021.01.011.

Yeo, N.C., Chavez, A., Lance-Byrne, A., Chan, Y., Menn, D., Milanova, D., Kuo, C.-C., Guo, X., Sharma, S., Tung, A., et al. (2018). An enhanced CRISPR repressor for targeted mammalian gene regulation. Nat. Methods *15*, 611–616.

Yiangou, L., Grandy, R.A., Morell, C.M., Tomaz, R.A., Osnato, A., Kadiwala, J., Muraro, D., Garcia-Bernardo, J., Nakanoh, S., Bernard, W.G., et al. (2019). Method to synchronize cell cycle of human pluripotent stem cells without affecting their fundamental characteristics. Stem Cell Rep. *12*, 165–179.

Stem Cell Reports, Volume *18*

Supplemental Information

Optimized whole-genome CRISPR interference screens identify ARID1A-dependent growth regulators in human induced pluripotent stem cells

Sunay Usluer, Pille Hallast, Luca Crepaldi, Yan Zhou, Katie Urgo, Cansu Dincer, Jing Su, Guillaume Noell, Kaur Alasoo, Omar El Garwany, Sebastian S. Gerety, Ben Newman, Oliver M. Dovey, and Leopold Parts

INDEX

Supplemental FIGURES 2

Supplemental Figure 1. 2

Supplemental Figure 2. 3

Supplemental Figure 3. 4

Supplemental Figure 4. 5

Supplemental Figure 5. 7

Supplemental Figure 6. 8

Supplemental Figure 7. 9

Supplemental Note 10

Supplemental Experimental Procedures 11

Supplemental Table 4: Primer sequences 16

Supplemental FIGURES

Supplemental Figure 1.

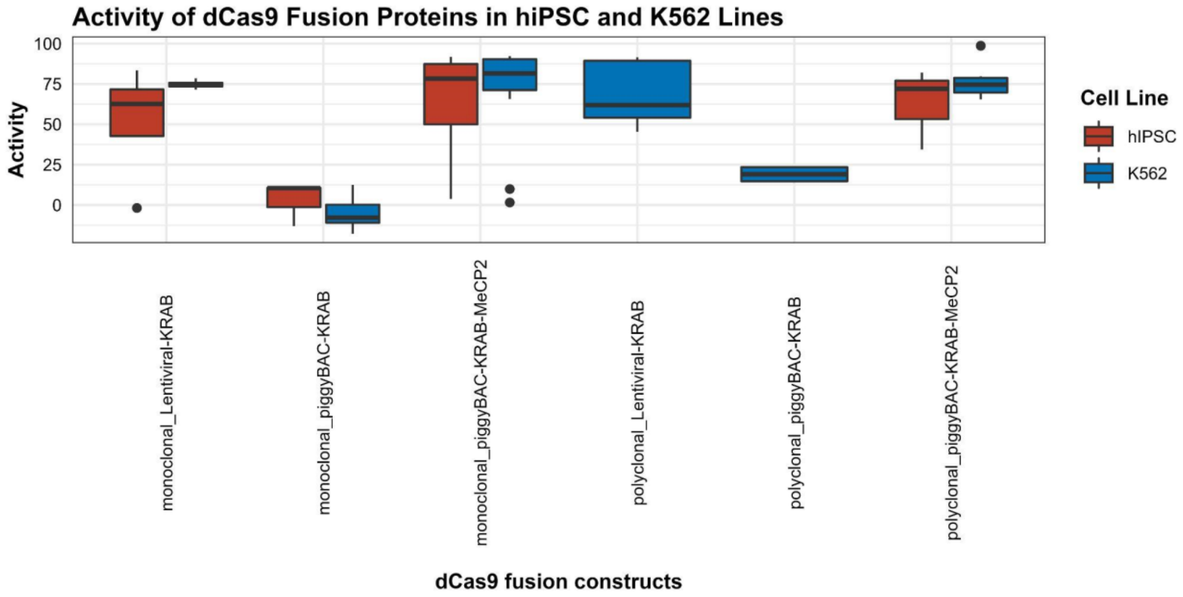

Supplemental Figure 1 – Related to Figure 1.C: Repression activity (y-axis) for different constructs and delivery methods (x-axis) into human iPSCs (red) and K562 cells (blue) for different mono- and polyclones (box and whiskers).

Supplemental Figure 2.

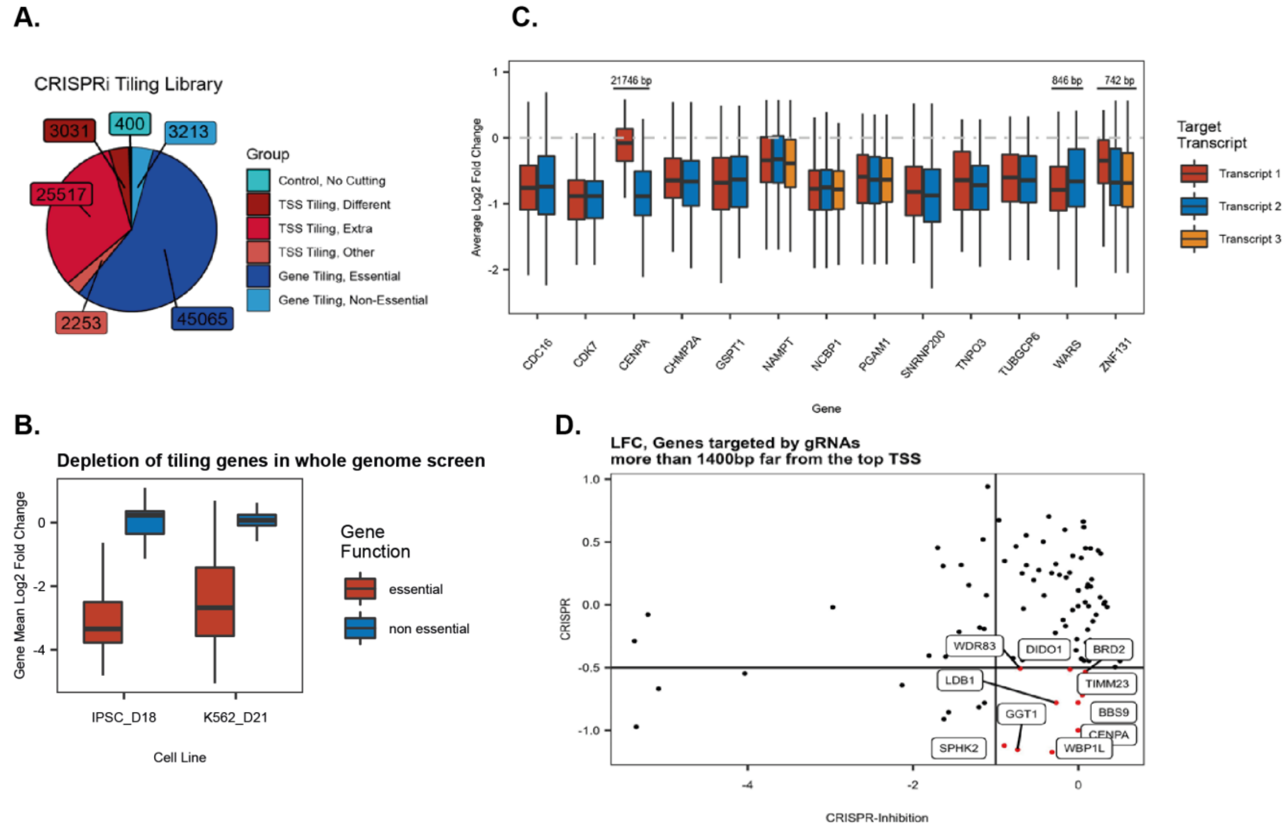

Supplemental Figure 2-(Related to Figure 2): **A.** Composition of CRISPRi tiling guide RNA library used in Figure 2. Numbers in boxes: guide RNA numbers in each sub-library. Teal: control library with no genomic targets. Red shades: transcription start site tiling library (“TSS Tiling, Extra”) tiling from 0 to +100bp of TSS site for the single transcript of 882 genes essential in human iPSCs; different TSS tiling library (“TSS Tiling, Different”) tiling -200 to +300 bp of TSS site for the top two transcripts of 20 essential genes for which the TSS annotation in human iPSCs did not match the canonical one; other transcript tiling library (“TSS Tiling, Other”) tiling top two transcripts of 20 essential genes. Blue shades: gene tiling library targeting all protospacer adjacent motifs in coding sequence of a single transcript of 451 Hart essential genes (blue; Hart et al., 2014) and 36 non-essential genes (teal). Contents of the libraries are provided in Supplemental Table 1. **B.** Gene-mean log2-fold change (y-axis) of genes in the transcription start site tiling library (Figure 2) in whole genome screen in iPSCs and K562 lines (x-axis). Box: median and quartiles; whiskers: 95th percentile; red: core essential genes, blue: non-essential genes. **C.** Average log2-fold change (y-axis) for 13 genes (x-axis) with multiple TSSs. Colors: gRNAs targeting the alternative TSSs. **D.** CRISPRi log2-fold change (x-axis) compared to CRISPR log2-fold change (y-axis) for genes (markers) that have a top TSS that is at least 1.4kb away from the targeted one.

Supplemental Figure 3.

A.

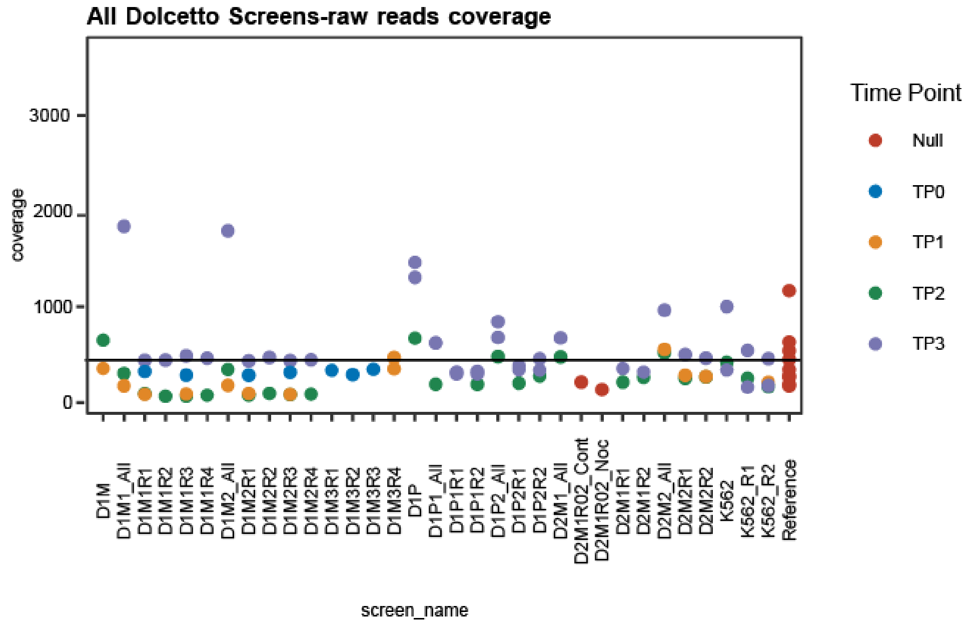

B.

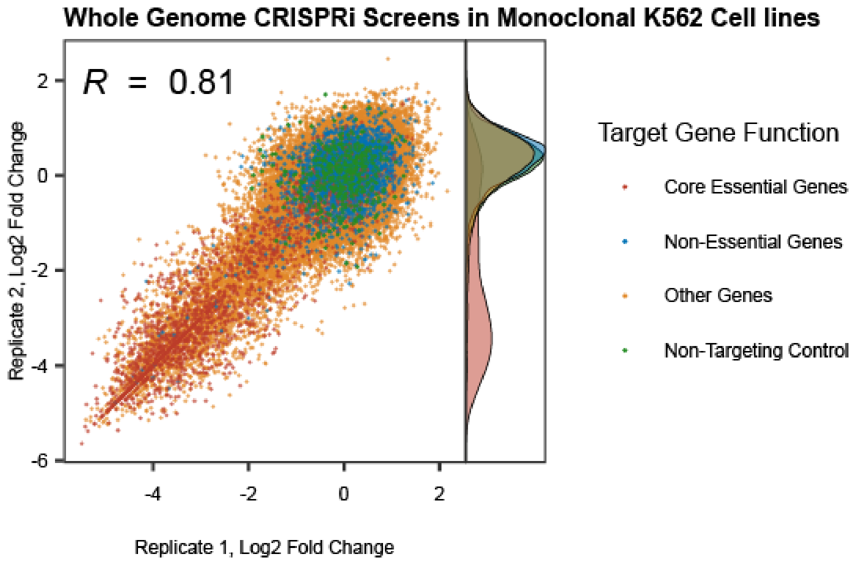

Supplemental Figure 3- (Related with Figure 3): **A.**Sequencing coverage (y-axis) of sequencing libraries (x-axis) of whole genome screens conducted using Dolcetto library (56,554 guide RNAs in total). Screen names; D: Donor, M: Monoclone, P: Polyclone, R: Replicate. Red: reference libraries, blue: timepoint 0 (days 3-5), yellow: timepoint 1 (days 9-11), green: timepoint 2 (days 13-15), purple: timepoint 3 (days 18-22), black line: average screen coverage (445X). **B.** Reproducibility of genome-wide screens in monoclonal K562 lines. gRNA log2-fold change in replicate 1 (y-axis) and replicate 2 (x-axis). Red: essential genes; blue: non-essential genes; yellow: other genes; green: non-targeting controls.

Supplemental Figure 4.

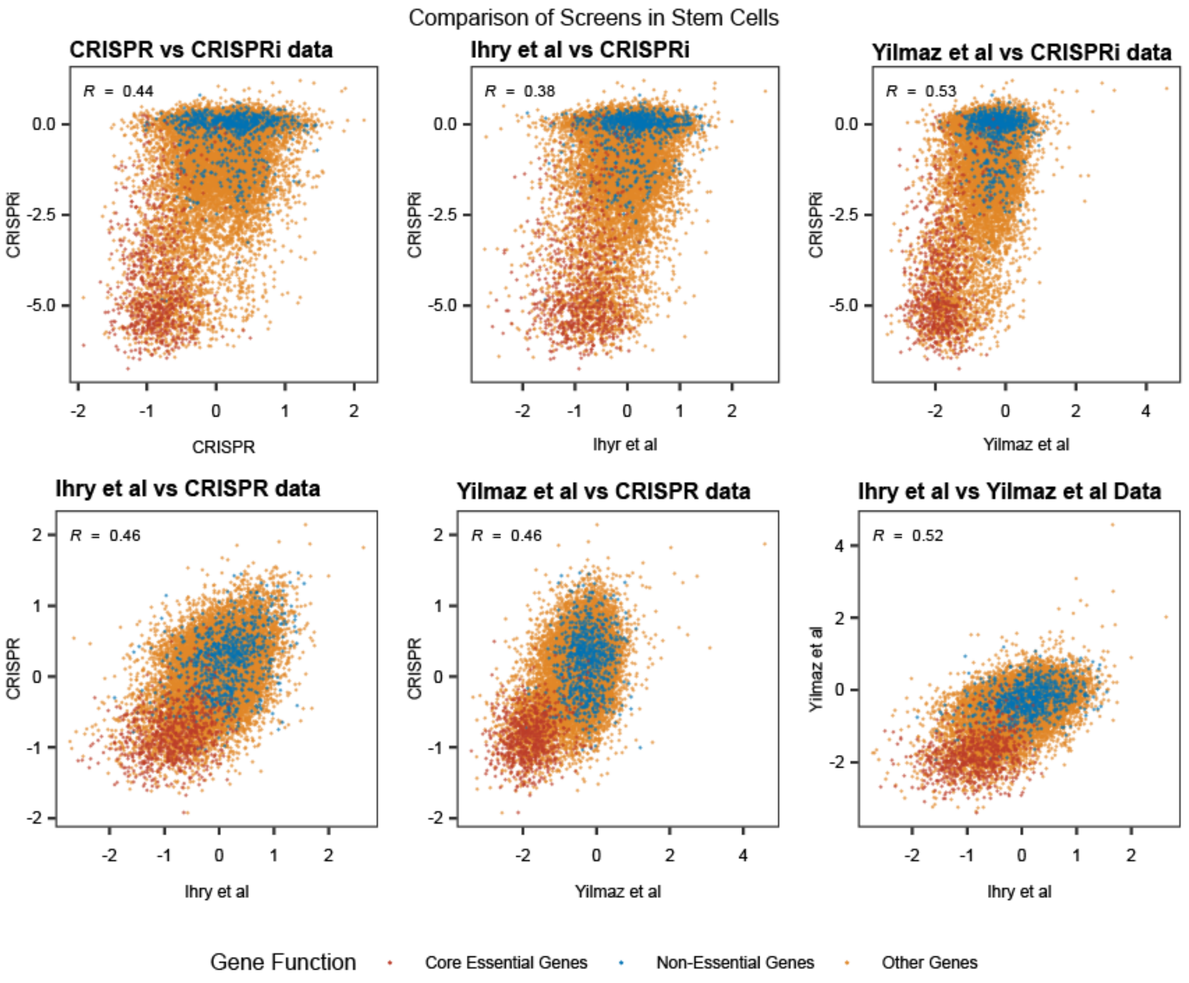

Supplemental Figure 4.A-(Related with Figure 4): Concordance of gene average log2-fold changes (x- and y-axes) of in-house CRISPR and CRISPRi screens in hiPSC with previously published CRISPR screens in diploid (Ihry *et al.*) and haploid (Yilmaz *et al.*) human embryonic stem cells (hESC). Red: essential genes; blue: non-essential genes; yellow: other genes. R: Pearson's correlation coefficient.

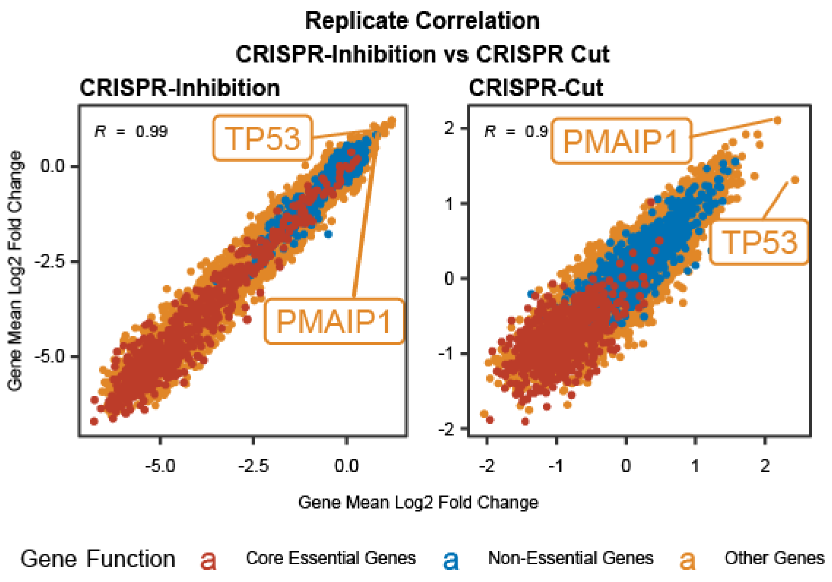

Supplemental Figure 4.B-(Related with Figure 4): Reproducibility and p53 effect in whole genome CRISPRi (left) and CRISPR (right) screens in monoclonal hiPSC lines. Gene mean log2 fold change value for replicate 1 (x-axis) and replicate 2 (y-axis). Data points for p53 and PMAIP1 genes are shown with an arrow. Red: essential genes; blue: non-essential genes; yellow: other genes.

Supplemental Figure 5.

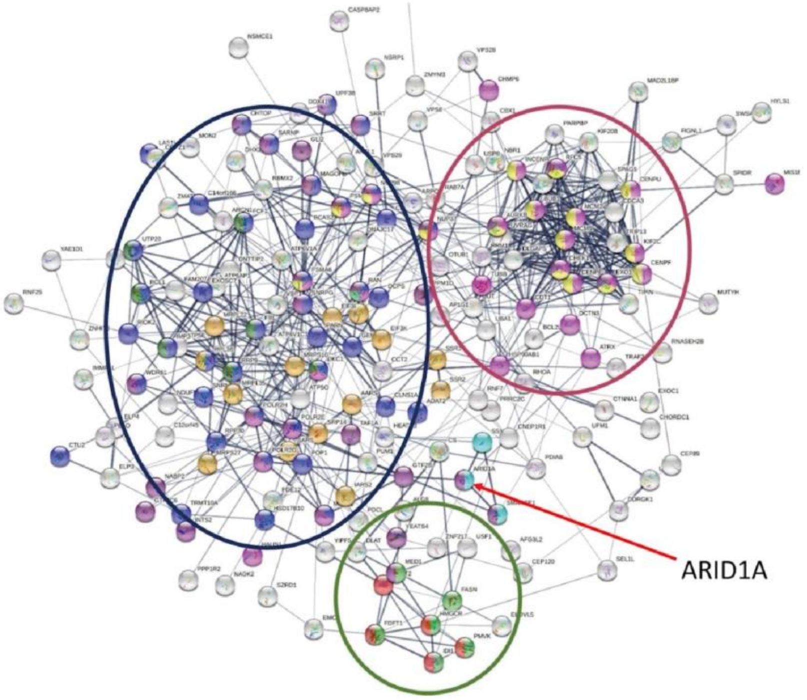

	Pathway	Description	Count in network	Strength	FDR
	HSA-8953854	Metabolism of RNA	41 of 659	0.82	4.3E-17
	HSA-1640170	Cell Cycle	30 of 647	0.69	1.3E-08
	HSA-72766	Translation	14 of 289	0.71	0.0012
	HSA-1655829	Regulation of cholesterol biosynthesis by SREBP (SREBF)	7 of 55	1.13	0.0015
	HSA-6790901	rRNA modification in the nucleus and cytosol	7 of 60	1.09	0.0022
	HSA-191273	Cholesterol biosynthesis	5 of 25	1.32	0.0049
	HSA-74160	Gene expression (Transcription)	31 of 1455	0.35	0.0124
	HSA-69620	Cell Cycle Checkpoints	16 of 269	0.8	5.7E-05
	GO:0071564	npBAF complex	3 of 12	1.42	0.0184

Supplemental Figure 5-(Related with Figure 5.E). STRING analysis of ARID1A. Large ellipses: manually curated clusters.

Supplemental Figure 6.

A.

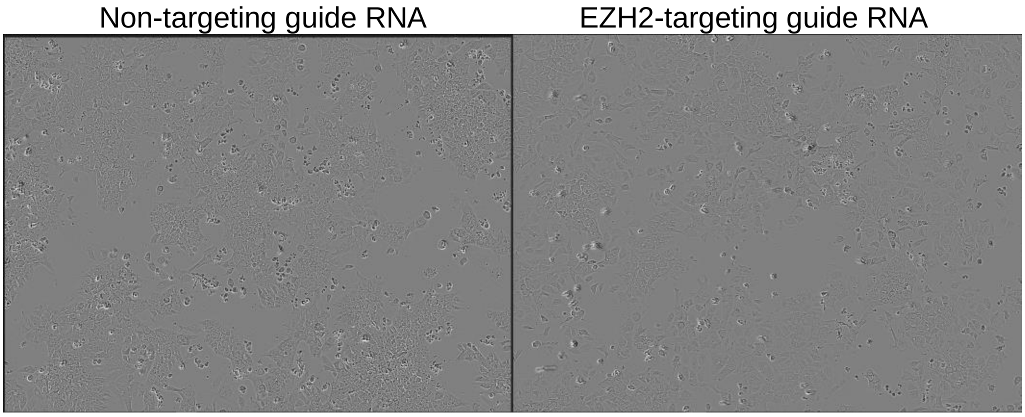

B.

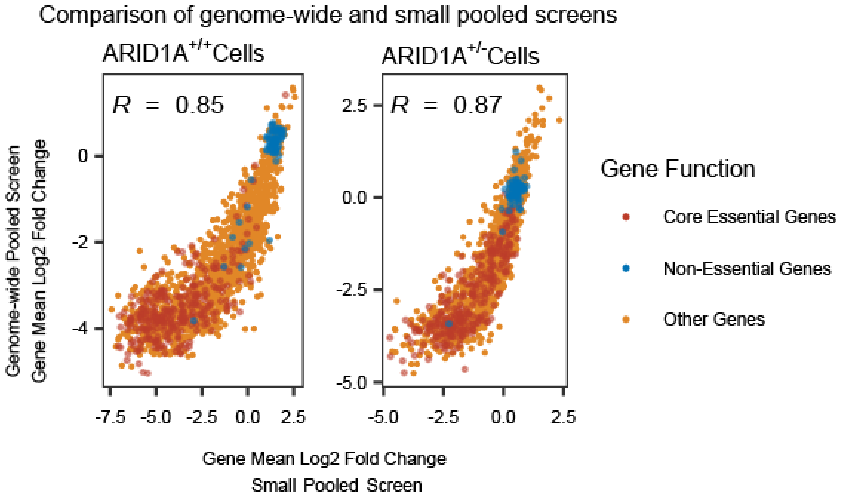

Supplemental Figure 6-(related with Figure 6). A. Microscope images from Incucyte live cell imaging system (10X objective) of ARID1A$^{+/+}$ hiPSCs infected with non-targeting guide RNA (left) and a guide RNA targeting EZH2 gene (right). **B.** Reproducibility of gene average log2-fold change (markers) in genome-wide (y-axis) and small-pooled screens (x-axis) in ARID1A$^{+/+}$ (left panel) and ARID1A$^{+/-}$ (right panel) hiPSC lines. Red: essential genes; blue: non-essential genes; yellow: other genes.

Supplemental Figure 7.

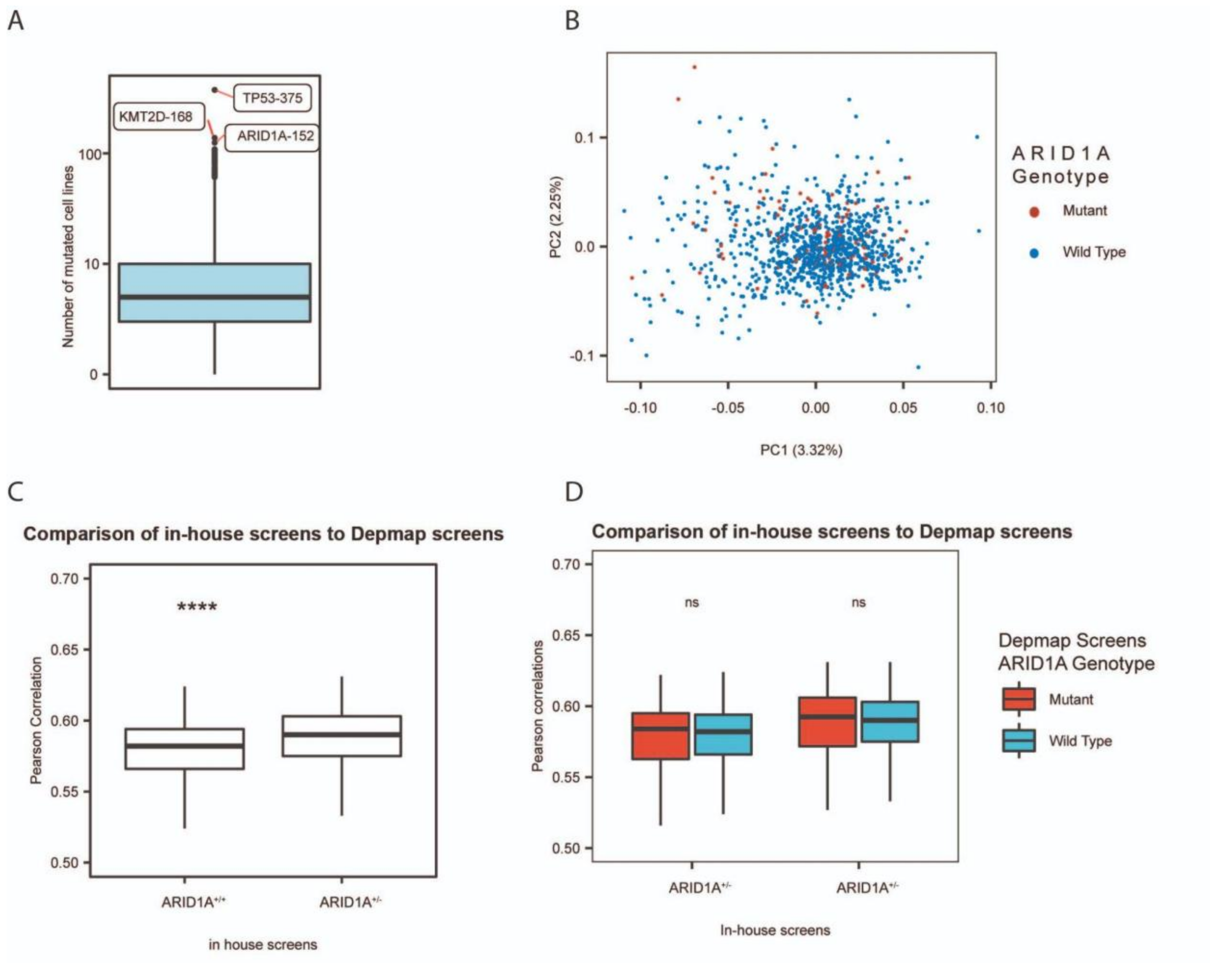

Supplemental Figure 7-(related with supplemental note): Comparison of in-house CRISPRi screens in ARID1A+/- and ARID1a+/+ hiPSC lines to DepMap screens. **A.** Number of mutated cell lines (y-axis) per gene. Labels: Gene and number of unique cancer lines with at least one mutation in a particular gene. Box: median and quartiles; whiskers: 95th percentile. **B.** PCA plot of DepMap CRISPR Gene Effect scores labeled according to ARID1A genotype of the cell line. Red: ARID1A mutant lines, Blue: ARID1A wild type lines. **C**. Correlation of in-house CRISPRi screens' Bayes Factor values to DepMap CRISPR Gene Effect scores. ARID1A$^{+/+}$: CRISPRi screen in ARID1A$^{+/+}$ hiPSC line, ARID1A$^{+/-}$: CRISPRi screen in ARID1A$^{+/-}$ hiPSC line. **D.** Correlation of in-house CRISPRi screens' Bayes Factor values to DepMap CRISPR Gene Effect scores separated by ARID1A genotype of DepMap cancer lines; Red: DepMap cancer lines with a mutation in ARID1A Gene. Blue: DepMap Cancer lines without ARID1A mutation. ARID1A$^{+/+}$: CRISPRi screen in ARID1A$^{+/+}$ hiPSC line, ARID1A$^{+/-}$: CRISPRi screen in ARID1A$^{+/-}$ hiPSC line.

Supplemental Note

Since our results in whole genome screens show gene interactions important for carcinogenesis, we asked if the sensitizing effect of the ARID1A mutation is similar to those of the damaging mutations in the cancer cell lines. To answer this question, we compared the survival of cancer cell lines upon gene perturbation in ARID1A wild-type and mutant contexts using data from the DepMap project (Dempster et al., 2021; Pacini et al., 2021). Among the 1,755 cancer lines in the database, 152 have at least one damaging mutation in the ARID1A gene (data: Q1/2022), making it one of the most mutated genes in this set of cancer lines (Supplemental Figure 7A). However, ARID1A mutation status was not associated with broad changes in gene essentiality in general (Supplemental Figure 7B). We did observe that gene essentiality estimates from our screen in the ARID1A+/- hiPSC line are significantly more correlated to the ones in cancer cell lines compared to the wild-type line (Supplemental Figure 7C). Still, this effect is small, and the correlation is not affected by ARID1A mutation status of the cancer line (Supplemental Figure 7D).

Supplemental Experimental Procedures

Lentivirus production. Lentivirus preps were produced in 293FT cells transfected with lentiviral delivery vectors together with second generation packaging system consisting of psPax2 (Addgene 12260), and pMD2.G (Addgene 12259) using Lipofectamine LTX with plus reagent (ThermoFisher 15338-030). 293FT cells were seeded on gelatin-coated 10cm culture dishes and grew until 80% confluent. On the day of transfection, 293FT medium was replaced by fresh medium. To prepare the transfection mix, 5.4 µg of a lentiviral vector, 5.4 µg of psPax2 (Addgene 12260), 1.2 µg of pMD2.G (Addgene 12259) and 12 µl of PLUS reagent were added to 3 ml of Opti-MEM Reduced Serum Media (ThermoFisher 31985-062) and incubated for 5 minutes. After addition of 36 µl of LTX reagent the mixture was incubated for another 30 minutes. The transfection mixture was added dropwise on top of 293FT cells and incubated at 37°C. After 48 hours, the medium with viral particles was collected and fresh medium was added on top of cells. After 24 hours, virus particles were harvested for the second time with medium, and added to the first harvest. All of the harvested virus was filtered using 0.45 um SFCA syringe filter (Nallgene, 190-2545), aliquoted and stored at -80ºC.

hIPSC infection. hiPSC cells were harvested using accutase (Stemcell technologies, 07920) and resuspended in mTeSR-E8 Media (Stemcell technologies, 05990) supplemented with 10µM Rock inhibitor (Ri) Y-27632 (Stemcell technologies, 72304). 0.5M cells were aliquoted and mixed with 200µl virus suspension and seeded on one well of 6 well-plate (in replicate). After 24 hours medium with Rock inhibitor was removed and fresh medium without Ri was added on top of cells. After two days, the medium was changed with mTeSR-E8 medium supplemented with 10 µg/ml Blasticidin (TOKU-E, B001), and selection was continued for 10 days.

K562 infection. $2x10^6$ K562 cells were aliquoted into 10 ml supplemented RPMI medium in duplicate. 100 µl of virus suspension and Polybrene (hexadimethrine bromide, Sigma) at a final concentration of 8 µg/ml were added, and the cell suspensions were centrifuged at 1000g for 30 minutes, resuspended, and seeded in 75 ml culture flasks with an additional 15 ml top up with supplemented RPMI media. After 48 hours, cells were passaged into media supplemented with 15 µg/ml Blasticidin (TOKU-E, B001), and selection was continued for 10 days.

PiggyBac transposition for hiPSCs. hiPSCs were transfected with

pB-CAGGS-dCas9-KRAB-MeCP2 or pPB-dCas9-KRAB vectors together with mPBase transposase vector using TransIT®-LT1 Transfection Reagent (Mirus Bio,MIR2300) as recommended by the producer. In summary, cells were harvested with accutase, and resuspended in mTeSR-E8 media supplemented with 10µM Rock inhibitor (Ri) Y-27632 (Stemcell technologies, 72304). Prepare 0.5M cell/ml cell suspension in mTeSR+E8 +Ri media. 400 µl of Opti-MEM Reduced Serum Media (ThermoFisher 31985-062) was aliquoted in a tube and 4µg plasmid DNA (1.8 µg delivery vector, 1.8 µg mPBase, 0.4µg pCS2-GFP) was added. The transfection mixture was incubated at room temperature for 20 minutes, and added onto one well of 6-well plate (Corning, 3516) with 0.5ml of mTeSR-E8+Ri media. After incubating the transfection mix on the plate for another 5 minutes, 1ml of $0.5x10^6$ cell/ml cell suspension was added on top. After 24 hours, the medium was changed to remove Rock inhibitor, and after 48 hours, the medium was changed to mTeSR-E8 supplemented with 10 µg/ml Blasticidin (TOKU-E, B001). Antibiotic selection continued for 10 days.

PiggyBac transposition for K562 cells. Cells were transfected with

pB-CAGGS-dCas9-KRAB-MeCP2 or pPB-dCas9-KRAB vectors together with mPBase transposase vector using Lipofectamine LTX reagent as described above for 293FT cells. pCS2-GFP vector was used as transfection control and the ratio of delivery vector:

mPBas:pCS2-GFP was 1:1:0.2. Blasticidin selection started after 2 days and continued for 10 days.

Cloning the reporter system

To measure CRISPRi efficacy, we devised a reporter system expressing BFP, GFP, and a guide RNA targeting GFP promoter from the same transient expression vector (Figure 1A). First, the pCS2-GFP vector was linearised with HindIII, and a gBlock (#639) with guide RNA target site was cloned into the GFP promoter region with Gibson assembly (Gibson et al., 2009) to obtain pCS2_CMV_gRNAtarget_GFP vector as a backbone. Meanwhile, the pU6-gRNA5 vector was linearised with BbsI, and oligos with target (#641) and mock (#640) gRNA were cloned after the U6 promoter with Gibson assembly. The U6+gRNA block was amplified from this vector (primers #642 and #643) and cloned into the SapI digested pCS2-BFP vector. The U6+gRNA+BFP block was then amplified from this vector (primers #646 and #647), the PGK promoter was amplified from pKLV2-U6gRNA5(gGFP5)-PGKBFPGFP-W vector (primers #644 and #645), and both cloned into the 5.2kb backbone of pCS2>CMV-GFP-SV40pA><CMV-mRuby2-bGHpA< vector linearised with EcoRi+MluI with Gibson assembly to produce pCS2-iREP-GFP-PGK-BFP-U6-gRNA-iRep or pCS2-iREP-GFP-PGK-BFP-U6-gRNA-mock vectors (all in one reporter system). All Gibson assembly reactions were conducted using NEB Gibson assembly master mix (E2611L) according to the manufacturer's protocol. Assembled constructs were purified using Monarch PCR purification kit (NEB, T1030S) and used to electroporate into electrocompetent bacteria (NEB, C3020K) according to the manufacturer’s instructions.

Generating the CRISPRi tiling library

Cloning and titration. sgRNAs were synthesized as complex oligonucleotide pools with several sub-pools (Genscript). Subpools were amplified with PCR, and Gibson assembly homology sequences were added to sgRNA sequences in a second round of amplification (primers #745 and #746). The lentiviral backbone vector pKLV2-U6gRNA5(BbsI)-ccdb-PGKpuroBFP-W (AddGene: 67974) (Tzelepis et al., 2016) was linearised with BbsI. Amplified sgRNA pools were inserted into the vector backbone with Gibson assembly reaction using NEB Gibson assembly master mix (E2611L) according to the manufacturer's protocol. Assembled constructs were purified using Monarch PCR purification kit (NEB, T1030S). The assembled plasmid library was electroporated into electrocompetent bacteria (NEB, C3020K) according to the manufacturer’s instructions as 3 reactions. 5µl of recovered bacteria was diluted 1:10 three times, and dilutions were seeded on ampicillin plates, while the remaining bacteria were seeded in liquid culture with ampicillin selection (100 µg/ml). The next day, bacterial colonies on agar plates were counted to calculate the coverage of the library, which was between 80x and 100x. The plasmid library pool was isolated from overnight bacteria culture using QIAGEN HiSpeed Plasmid Midi (small pooled library) of maxi (TSS tiling library) kits (QIAGEN, 12643 and 12662). Lentivirus was produced from cloned plasmid pools in 293FT cells as described above. To determine virus titer hiPSC-(Fiaj-1) dCas9-KRAB-MeCP2 cells were suspended as 0.16M cells/ml suspension. 5 x 2.5 ml of cell suspensions were mixed with five different amounts of virus between 20µl to 100µl. 1ml of each virus cell mixture was seeded one well of vitronectin pre-coated 12 well plate. After 3 days, cells were collected with accutase, washed, and resuspended in FACS buffer (PBS with 2% FBS) and analysed by FACS (Cytoflex, BD). Best fit line formula of percentage BFP positive cells against virus volume was used to calculate the volume of virus required for 0.3 multiplicity of infection (MOI).

Reamplification and titration of the Dolcetto CRISPRi library

Dolcetto library (Sanson et al., 2018) was acquired from Addgene (#1000000114) as two plasmid pools, each with a half-library (Set A and B) in the XPR_500 backbone. Only Dolcetto Set A plasmid pool was used in this study. 350 ng Dolcetto setA library pool was used to transform 100 µl electrocompetent bacteria (NEB, C3020K), split into 4 x 25 µl

electroporations. Electroporated bacteria were recovered in 3ml recovery media and all 4 cultures were mixed together. 5 ul of the culture mix was diluted 1:10 serial diluted 5 times and each dilution seeded on LB agar+ Amp plates. Remaining transformed bacteria culture seeded in 0.5ml LB+ Ampicillin (100 µg/ml) was left overnight. Plasmid library pool was isolated from overnight culture using HiSpeed maxi plasmid isolation kit (QIAGEN, 12662). Lentivirus was produced from the amplified plasmid pool as described above. To titrate the virus for hiPSCs, hIPSC+dCas9_KRAB+MeCP2 lines were collected with accutase and 1 M cells were resuspended in mTeSR-Plus +Ri media at $1.3x10^5$ cells/ml. Different volumes of virus prep from 0 µl to 1.6µl were mixed with mTeSR-Plus +Ri media up to 600 µl. 600 µl of cell suspension was added on top of each virus dilution and mixed. 50 µl of each virus cell mixture were seeded into 10 wells of each of 96-well plates, resulting in two plates with 6 different virus/cell ratios. On day 2 post-infection, the medium in one plate was changed with mTeSR-Plus, and in the other plate with mTeSR-Plus + Puromycin (0.5 µg/ml). On day 4 post-infection, the medium in each well was replaced by 100µl mTeSR-Plus medium + 20µl MTS dye (Promega, G3582) to compare living cell amounts between plates. The plates were incubated at 37 C for 2 hours, followed by the addition of 25µl of 10% SDS solution on each well. Plates were analyzed in Multiskan™ GO Microplate Spectrophotometer plate reader (Thermo Scientific, 51119200) at wavelengths of 490 nm and 700 nm. Raw reads were adjusted after blank and background (700 nm) readings were subtracted from target readings (490 nm). Virus titer was determined from the ratio of puromycin treated and untreated cells. To titrate the virus for K562 cells, the cells were collected, counted, and aliquoted to have 50,000 cells per well, 12 wells for each of 6 virus titers. Different amounts of virus prep were mixed with cell suspensions and distributed on a 96-well plate. Each well was topped up with supplemented RPMI media + Polybrene (8ug/ml final concentration) up to 150 ul. Plates were centrifuged at 1000 rcf for 30 minutes and resuspended. On day 3 after infection, cells were split into two 96-well plates. Cells in one plate were resuspended in supplemented RPMI media and the other plate in supplemented RPMI + puromycin (2µg/ml) media. On day 6 after the infection relative amounts of cells in each well were determined by MTS assay as described above.

Genomic DNA isolation and sequencing library preparation

Aliqouted cell pellets were thawed at room temperature and resuspended in 100 mM Tris-HCl, pH 8.0, 5 mM EDTA, 200 mM NaCl, 0.2% SDS and 1 mg/ml Proteinase K. After overnight incubation at 55oC, RNase was added on top of the cell suspension to the final concentration of 10 µg/µl, followed by 3h incubation at 37oC. Genomic DNA was precipitated with 100% isopropanol, spooled out, washed in 70% EtOH and air dried at room temperature. Following resuspension in TE buffer overnight, DNA was quantified with Quant-iT Broad Range kit (Q33130, ThermoFisher). gRNA cassettes were amplified from genomic DNA with two consecutive PCR reactions taking the target library coverage into account (used genomic DNA corresponding cell number for 500x for final timepoints, minimum 250x for interval timepoints). The first reaction with Q5 Hot Start High-Fidelity 2X Master Mix (NEB) amplifies the gRNA cassette (primers #1 and #2 for CRISPRi and small pooled screen libraries, primers #1 and #638 for Dolcetto library). PCR reactions were purified with QIAquick PCR Purification Kit (Qiagen, 28106) and quantified with nanodrop. Each library was diluted to 1ng/µl and used as template for the second PCR reaction where sequencing adaptors with index sequences were added as described before (primers #15 and #NN indexing) (Tzelepis et al. 2016). PCR reactions were purified with 0.7X AMPure XP beads (Agencourt AMPure XP beads; Beckman, Cat.no. A63881), quantified with Quant-it High Sensitivity Kit (Q33120, ThermoFisher), pooled and single end sequenced using primer #16.

Arrayed gRNA cloning, lentivirus production

Cloning. Each guide RNA was ordered as top and bottom strand oligos mixed in single well creating double stranded DNA with cloning overhangs (Sigma Aldrich, Supplemental Table 4).

Oligos were phosphorylated with T4 Polynucleotide Kinase (NEB M0201) at 37oC for 30 minutes. pKLV2-U6gRNA5(BbsI)-PGKpuro2AmCherry-W (Addgene 67977) (Tzelepis et al. 2016) was linearised with BbsI at 37oC overnight. Phosphorylated gRNA oligos were cloned into linearized backbone with T4 DNA ligase (NEB M0202*) by incubating 1h at room temperature. 3 µl of the ligation products were used to transform 20µl of zymo mix&go 10B competent cells (Zymo Research, T3020). Transformed cells were grown in 2ml 2XLB media overnight. gRNA plasmids were isolated using QIAprep Turbo Miniprep Kit (Qiagen, 27191). gRNA sequences were confirmed by Sanger sequencing.

Lentivirus production. Flat bottom 96 well plates were coated with 0.1% gelatin, and 293T cells were seeded as 20K cells/well. 0.1ug LV transfer plasmid, 0.1ug psPAX2, and 0.02 ug pMD2.G were used to transfect cells with Lipofectamine LTX Reagent (15338100, Thermo Fisher) by scaling manufacturer's protocol. Cultures were collected from each well after 48 hours, centrifuged at 500 rcf for 20 minutes, and supernatant was collected carefully without disturbing the pellet.

Cell phenotyping

Comparison of WT and ARID1A$^{+/-}$ lines. Wild type and ARID1A$^{+/-}$ Kolf hiPSC lines were seeded at 20,000/cm2 density in 12-well plates (6 well each) in mTeSR Plus medium supplemented with Rock inhibitor (Ri). The next day, medium was changed to remove Ri and the plate was placed in Incucyte S3 live imaging system (Sartorius) and imaged every 2 hours for 50 hours using 10x objective taking 16 frames per well. The growth rate was measured as percent confluency and normalized to the first time point.

Oprozomib treatment measurements. Wild type and ARID1A$^{+/-}$ Kolf hiPSC lines were seeded at 20,000/cm2 and 30,000/cm2 density respectively in 12 wells of 24 well plate. After 36 hours, both lines were at approximately 25% confluence. At this point, medium was replaced with mTeSR-Plus supplemented with different concentrations of Oprozomib (APExBIO, A1937) (10uM, 2uM, 0.4uM, 0.08uM, 0,02uM). The plate was then placed in the Incucyte live imaging system, and imaged every 2 hours for 26 hours using 10x objective taking 16 frames per well. At each time point, the growth rate was measured as percent confluency, and normalised to the first time point. Confluence was compared to no treatment wells at 32h after seeding.

CRISPR vs CRISPRi comparison. Monoclonal Fiaj-1_dCas9-KRAB-MeCP and Fiaj-1_Cas9 lines were collected, counted and infected with targeting and non-targeting guides (Supplemental Table 3). Infected cells were seeded on two wells of 24 plates and monitored in Incucyte S3 Live Cell imaging System with 10x objective taking 16 frames per well every two hours. Cell death was determined as the decrease in confluency after media change.

Growth assays with live imaging system. Cell growth was measured in Incucyte S3 Live Imaging System with confluence as a measure of cell number in different assays. Additionally, to validate confluence vs cell number correlation, cells were seeded in a 96 well plate at different densities and cell growth was measured by taking images every two hours with a 10X objective and 5 frames per well for 24h. After final measurement of confluence in the Incucyte live imaging system for each, we compared the optical density (OD) value obtained to the MTS assay and found it to be highly correlated.

.

Cell staining and FACS. Wild type and ARID1A+/- lines were infected with lentivirus with EZH2 targeting guides and seeded in 12 well plates. Infected cells were selected with puromycin for 9 days. Cells were collected with accutase and counted. 5x106 cells were aliquoted and washed once with FACS buffer (5% FBS in PBS). Cells were fixed in 1% PFA for 30 minutes at room temperature and blocked in FACS buffer for another 30 minutes. 1x106 cells were aliquoted in tubes and incubated with stem cell marker, perCP-CY-5.5-Mouse antihuman-TRA-1-60 antibody (BD Pharmingen, 561573) for 1 hour at room temperature.

HEK293T cells were used as a negative control for antibody sensitivity. Stained cells were analysed in CytoFLEX flow cytometer and with FlowJo analysis software (Beckman Coulter).

Supplemental Table 4: Primer sequences

Primer	Sequence	Name
#639	TGCCTGGGGACGTCGGAGCATACCTCATCAGGAACATGTTGGTGATTTAGGTGACACTATAGAATACAAGCT	Gibson-target-iREP
#640	GCTTTATATATCTTGTGGAAAGGACGAAACACCGGTTAAGCGACTTCGGCCAGGTTTAAGAGCTATGCTGGAAACAGCA	Gibson-mock-grna
#641	GCTTTATATATCTTGTGGAAAGGACGAAACACCGTACCTCATCAGGAACATGTGTTTAAGAGCTATGCTGGAAACAGCA	Gibson-iREP-gRNA
#642	GGGCCTCTTCGCTATTACGCCAGACGCGTCCAAGGTCGGGCA	GA-pCS2-U6
#643	ATACGCCATATTGAATTGGCTATGGTCGACACTAAAGGGAACAAAAGCGGATCC	GA-pCS2-scaffold
#644	CCGCCAGTGTGATGGATATCTGCAGTTAATTTAGCTTGTGCCCCAGTTTGCT	GA-BFP-bGHpA
#645	CTTCCTGCCCGACCTTGGACGCGTAATTCTACCGGGTAGGGGAGGCGC	GA-U6-PGK
745	GGCTTTATATATCTTGTGGAAAGGACGAAA	ssoligo-PCR-F
746	ACTTGCTATGCTGTTTCCAGCATAGCTCTT	ssoligo-PCR-R
#1	ACACTCTTTCCCTACACGACGCTCTTCCGATCTCTTGTGGAAAGGACGAAACA	Sequencing_gRNA_library_amplification
#2	TCGGCATTCCTGCTGAACCGCTCTTCCGATCTCTAAAGCGCATGCTCCAGAC	Sequencing_gRNA_library_amplification
#638	TCGGCATTCCTGCTGAACCGCTCTTCCGATCTTCTACTATTCTTTCCCCTGCACTGT	Dolcetto-lib-R
#15	AATGATACGGCGACCACCGAGATCTACACTCTTTCCCTACACGACGCTCTTCCGATCT	Sequencing_indexing_PCR_indexing
#NN	CAAGCAGAAGACGGCATACGAGATN11GAGATCGGTCTCGGCATTCCTGCTGAACCGCTCTTCCGATCT	Sequencing_indexing_PCR
#16	TCTTCCGATCTCTTGTGGAAAGGACGAAACACCG	Sequencing
